# Biogeographic Distribution Patterns of Bacteria in Typical Chinese Forest Soils

**DOI:** 10.3389/fmicb.2016.01106

**Published:** 2016-07-13

**Authors:** Zongwei Xia, Edith Bai, Qingkui Wang, Decai Gao, Jidong Zhou, Ping Jiang, Jiabing Wu

**Affiliations:** Institute of Applied Ecology, Chinese Academy of SciencesShenyang, China

**Keywords:** Chinese forest soil, microbial biogeography, soil bacterial diversity, soil pH, 16S rRNA, high throughput sequencing

## Abstract

Microbes are widely distributed in soils and play a very important role in nutrient cycling and ecosystem services. To understand the biogeographic distribution of forest soil bacteria, we collected 115 soil samples in typical forest ecosystems across eastern China to investigate their bacterial community compositions using Illumina MiSeq high throughput sequencing based on 16S rRNA. We obtained 4,667,656 sequences totally and more than 70% of these sequences were classified into five dominant groups, i.e., *Actinobacteria, Acidobacteria, Alphaproteobacteria, Verrucomicrobia*, and *Planctomycetes* (relative abundance >5%). The bacterial diversity showed a parabola shape along latitude and the maximum diversity appeared at latitudes between 33.50°N and 40°N, an area characterized by warm-temperate zones and moderate temperature, neutral soil pH and high substrate availability (soil C and N) from dominant deciduous broad-leaved forests. Pairwise dissimilarity matrix in bacterial community composition showed that bacterial community structure had regional similarity and the latitude of 30°N could be used as the dividing line between southern and northern forest soils. Soil properties and climate conditions (MAT and MAP) greatly accounted for the differences in the soil bacterial structure. Among all soil parameters determined, soil pH predominantly affected the diversity and composition of the bacterial community, and soil pH = 5 probably could be used as a threshold below which soil bacterial diversity might decline and soil bacterial community structure might change significantly. Moreover, soil exchangeable cations, especially Ca^2+^ (ECa^2+^) and some other soil variables were also closely related to bacterial community structure. The selected environmental variables (21.11%) explained more of the bacterial community variation than geographic distance (15.88%), indicating that the edaphic properties and environmental factors played a more important role than geographic dispersal limitation in determining the bacterial community structure in Chinese forest soils.

## Introduction

Microbes are widely distributed in soils and play a very important role in nutrient cycling and ecosystem services. It is generally recognized that the microbial diversity and composition are key determinants of their ecological functions (Brussaard, 1997). Many studies in the recent decade have shown that soil microbes from various ecosystems exhibit biogeographic distribution patterns (Fierer and Jackson, 2006; Ge et al., 2008; Lauber et al., 2009; Chu et al., 2010), which generally differs from the patterns observed for plant and animal taxa (Levin, 1992; Gaston, 2000; Allen et al., 2002). The biogeographic distribution patterns of animals and plants are simultaneously determined by both environmental heterogeneity and geographic dispersal limitation (Ganderton and Coker, 2005; Lomolino et al., 2006), while the biogeographic distribution patterns of soil bacteria is thought to be mainly determined by soil variables and local environment conditions (Garbeva et al., 2004; Ramette and Tiedje, 2007; Green et al., 2008). However, due to the limitations by the technique of high-resolution classification and the difficulties in large-scale survey, our understanding of the biogeographic distribution of soil bacterial community remains limited (Martiny et al., 2006).

Previous studies on the biogeographic distribution of bacterial communities indicated that soil bacterial community structure was influenced by edaphic, climatic or land cover characteristics (Cho and Tiedje, 2000; Zhou et al., 2002; Yergeau et al., 2007), and the controlling factors varied at different spatial scales and in different ecosystem types. Dequiedt et al. (2009) reported that the bacterial community composition was more related to soil properties and land cover than to climatic and geomorphologic characteristics in four different regions of France. In contrast, soil bacterial community abundance and structure in arid and semiarid regions were found to be significantly correlated to both precipitations (or expressed as aridity) and soil properties at large spatial scales (Pasternak et al., 2013; Maestre et al., 2015; Wang et al., 2015). Among the soil characteristics, soil pH was often reported as an overriding factor on determining bacterial communities structure (Fierer and Jackson, 2006; Baker et al., 2009; Liu et al., 2014) and some individual taxonomic groups (Nicol et al., 2008; Davis et al., 2009; Jenkins et al., 2009; Jones et al., 2009). Additionally, other parameters have also been found to influence the composition and diversity of soil bacterial communities, such as soil nutrient availability (Broughton and Gross, 2000; Liu et al., 2010; Naether et al., 2012), salinity (Crump et al., 2004; Lozupone and Knight, 2007), plant diversity and community composition (Stephan et al., 2000; Wardle et al., 2004). Although the same factor may show different levels of influences on bacterial community structure in different ecosystems or at different spatial scales, some common bacteria may exist in many areas. For example, the *Verrucomicrobia* phylum was detected in almost all soils collected across a range of biomes in North America, South America, Europe, and Antarctica (Bergmann et al., 2011). Therefore, it is important to understand the distribution patterns of main bacterial groups at different spatial scales and at the same time explore the factors determining these patterns.

In contrast to the relatively large number of studies examining soil bacterial community structure across broad spatial ranges in Europe and the Americas, a comprehensive understanding of the biogeographic distribution of soil bacterial community across China is still lacking. Until now, only a few studies have focused on the soil bacterial community structure at a large scale in China (Liu et al., 2014; Wang et al., 2015). In this study, we collected 115 soil samples in forests from north to south China, covering tropical forest, subtropical forest, temperate forest and boreal forest types, and investigated on the spatial variations of these soil bacterial community compositions. Soil bacterial communities were determined based on the data of 16S rRNA sequences (V4 – V5 hypervariable region) using Illumina MiSeq. The objectives of this study were: (1) to determine the composition of the soil bacterial community of different forest types across eastern China; (2) to explore the biogeographic distribution patterns of soil bacterial communities across such a wide range; and (3) to examine the dominant factors in shaping the distribution of the bacterial community structure of these soils.

## Materials and Methods

### Site Selection and Soil Sampling

**FIGURE 1:**
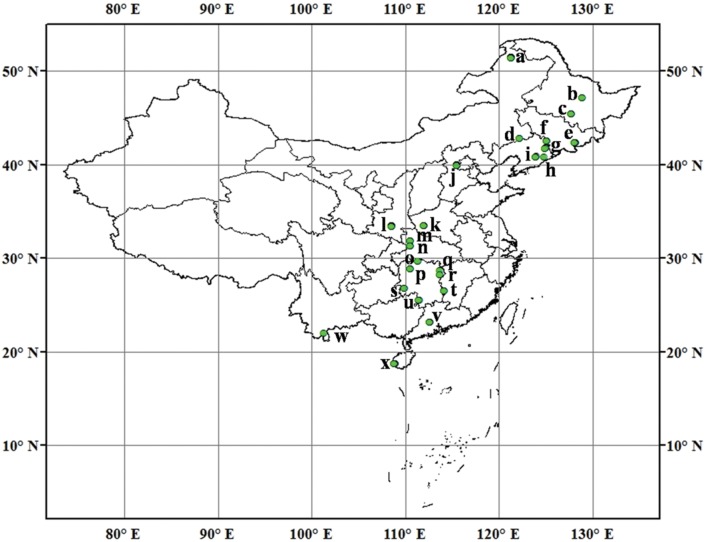
**Sampling locations and sample number in each location across North and South of Chinese forest soils.** (a) Daxingan, 12; (b) Liangshui, 2; (c) Maoershan, 4; (d) Daqinggou, 2; (e) Changbaishan, 6; (f) Binglashan, 4; (g) Qingyuan, 5; (h) Baishila, 1; (i) Caohekou, 4; (j) Beijing, 12; (k) Baotianman, 9; (l) Qinling, 12; (m) Shennongjia, 3; (n) Huanglianba, 3; (o) Changde, 2; (p) Huaihua, 3; (q) Yueyang, 1; (r) Changsha, 2; (s) Huitong, 3; (t) Zhuzhou, 3; (u) Yongzhou, 3; (v) Dinghushan, 9; (w) Xishuangbanna, 1; (x) Jianfengling, 9.

We collected 115 mineral soil samples from typical forests with different vegetation types across north and south China with the latitudes ranging from 18.70°N to 51.53°N (**Figure 1**). These soil samples harbor a wide range of soil types and edaphic and environmental characteristics (**Supplementary Table S1**). Mean annual temperature (MAT) and mean annual precipitation (MAP) data in sampling locations were obtained from WorldClim^1^ All samples were taken in July and August, 2014. At each site, eight to ten randomly selected soil cores (0–10 cm, 5 cm in diameter) were collected within an area of about 400 m^2^. Soil samples were combined into one composite sample for each site, and then transported at 4°C to the Institute of Applied Ecology, Chinese Academy of Sciences at Shenyang, China. Soil samples were sieved through 2-mm mesh to thoroughly homogenize and remove roots, plant detritus and stones. A portion of each soil sample was stored at -20°C until DNA extraction. The remaining soils were used to determine extractable 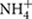 and 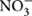 contents, soil microbial biomass carbon (MBC) content and soil physicochemical properties.

### Soil Physicochemical Properties

Soil pH was measured using a pH meter in the supernatant after shaking soil – water (1:5 w/v) mixture for 30 min. Soil total carbon, total nitrogen were determined using an Elemental analyzer (VarioEL III, Germany), while soil available phosphorus and total phosphorus were determined as previously described methods (Kuo, 1996). Soil 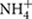 and 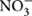 were extracted with 2 M KCL solution for 1 h on a shaker, and their contents were determined using a flow injection analyzer (Futura, Alliance, France). The soil MBC was estimated using the chloroform fumigation-extraction method (Vance et al., 1987; Joergensen, 1996). The soil exchangeable K^+^, Na^+^, Ca^2+^, and Mg^2+^ were determined by extracting the soils with ammonium acetate (Thomas, 1982). Amounts of Ca^2+^ and Mg^2+^ in the extracts were analyzed by atomic absorption spectrometry (AAS) and K^+^ and Na^+^ were analyzed by flame photometry.

### Soil DNA Extraction

Each soil DNA was extracted from the 0.25 g freeze-dried soil after sampling using a Mobio PowerSoil DNA Isolation Kit (MoBio Laboratories, Carlsbad, CA, USA) according to the manufacturer’s instructions. DNA was eluted with 100 μl Tris buffer (10 mM), quantified by spectrophotometer at 260 nm and stored at -20°C until use.

### Bacterial 16S rRNA Amplicon and Barcoded Sequencing

Soil DNA samples were sent to Novogene Company (Beijing, China) for high-throughput sequencing. The amplicon targeting V4-V5 hypervariable region of bacterial 16S rRNA was amplified with primer set 515F/806R, which contained sample specific 6-bp barcodes in the 5′ ends of them. The sequences of 515F and 806R were 5′- NNN NNN (barcode) GTG CCA GCM GCC GCG GTA A -3′ and 5′- NNN NNN (barcode) GGA CTA CHV GGG TWT CTA AT -3′, respectively. All PCR reactions were carried out in a volume of 30 μl mixture containing 15 μl of Phusion^®^ High-Fidelity PCR Master Mix (New England Biolabs), 0.2 μM of each primer, about 10 ng template DNA, and ddH_2_O filled to 30 μl. Thermal cycling included an initial denaturation at 98°C for 1 min, followed by 30 cycles of denaturation at 98°C for 10 s, annealing at 50°C for 30 s, and elongation at 72°C for 1 min, with a final extension at 72°C for 5 min. PCR products were detected by 2% agarose gel electrophoresis, and those with bright main strip between 400 and 450 bp were chosen for further experiments. Equal amounts of the PCR product from each sample were pooled and then purified with GeneJET Gel Extraction Kit (Thermo Scientific). The sequencing library was generated using NEB Next^®^ Ultra^TM^ DNA Library Prep Kit for Illumina (NEB, USA) following the manufacturer’s instructions and thus sequencing adapters were added to 5′ ends of amplicon. The library quality was assessed on the Qubit^@^ 2.0 Fluorometer (Thermo Scientific) and Agilent Bioanalyzer 2100 system. At last, the qualified library was sequenced on the Illumina MiSeq platform, producing 250 bp/300 bp paired-end reads.

### Processing of Sequencing Data

Paired-end reads from the original amplicon were merged using FLASH (Magoč and Salzberg, 2011) which is designed to merge paired-end reads when there are overlaps between reads1 and reads2. Paired-end reads was assigned to each sample according to the unique barcodes which were removed together with primers subsequently. Sequences were analyzed using QIIME software package (Quantitative Insights Into Microbial Ecology^2^) (Caporaso et al., 2010), and in-house Perl scripts were used to analyze alpha-(within samples) and beta-(among samples) diversity. First, merged reads were filtered by QIIME quality filters. Then the clean sequences obtained with ≥97% similarity level were assigned to the same operational taxonomic units (OTUs). A representative sequence from each OTU was picked and annotated using the RDP classifier for taxonomic information (Wang et al., 2007) and aligned with “Core Set” in the GreenGene database for phylogenetic information (DeSantis et al., 2006). In order to unify the survey (Shaw et al., 2008), a subsample of randomly selected 7300 sequences in each sample was used for bacterial alpha-diversity (phylotype richness and phylogenetic diversity) and beta-diversity (community dissimilarity index) analyses. Observed Species and Phylogenetic Diversity Whole Tree (PD for short) indexes formed during alpha-diversity analysis were used to indicate phylotype richness and phylogenetic diversity in samples, respectively. Unifrac metric was used to compare the difference of overall community composition between each pair of samples (Lozupone and Knight, 2005), and thus generating unweighted and weighted pairwise unifrac distance matrixes. All sequences in this study are available in Sequence Read Achieve (SRA) database of NCBI under accession number SRP070864.

### Statistic Analyses

Correlation (Pearson’s rank correlation) or regression analysis between soil/site characteristics and individual phyla or between soil/site characteristics and indexes of community diversity and composition were performed in SPSS 17.0 for Windows. The other statistical analyses were conducted using the program R v.3.2.0 (R Development Core Team, 2006). The “Bray–Curtis” dissimilarity matrix for the bacterial community composition and the “Euclidean” dissimilarity matrices for geographic distance and environmental variables were constructed with the “vegdis” “function in the “vegan” package (Oksanen et al., 2016). The non-metric multidimensional scaling (NMDS) and cluster analysis of soil samples in the bacterial community composition was conducted with the “metaMDS” (Minchin, 1987) and “hclust” functions (Murtagh, 1985) based on the “Bray–Curtis” dissimilarity matrix within the package “vegan,” respectively. Additionally, we conducted 1 minus Unifrac distance (unweighted or weighted) in the total community structure to estimate the bacterial community similarity. BioEnv procedure (Clarke and Ainsworth, 1993) was performed to select the environmental variables which were further used to construct environmental distance matrix with the “vegdist” function. Using principle coordinates of neighbor matrices (PCNM) method (Borcard and Legendre, 2002), the geographic coordinates of the sites were transformed to significant vectors that could be used to construct geographic distance matrix across sites. Mantel tests with 999 permutations (Legendre and Legendre, 2012) were used to examine the correlation (Pearson’s rank correlation) between geographic or environmental distance and bacterial community distance within the vegan package. The canonical correspondence analysis (CCA) (Legendre and Legendre, 2012) was employed to identify the most important soil environmental factors shaping bacterial community structure. Monte Carlo permutation test (permutest) and “envfit” functions (Legendre et al., 2011) were used to test the significant environmental variables during CCA analysis. These significant PCNM vectors and environmental variables were used as explanatory variables in constrained ordinations (CCA) for variation partition analysis.

## Results

### Soil and Site Characteristics

The latitude of each sampling site was highly correlated with the site’s MAT (*r* = -0.982, *P* < 0.001) and MAP (*r* = -0.873, *P* < 0.001) (**Table 1**). Soil pH showed significant correlations with concentrations of three types of exchangeable cations, i.e., K^+^ (*r* = 0.685, *P* < 0.001), Ca^2+^ (*r* = 0.843, *P* < 0.001), Mg^2+^ (*r* = 0.776, *P* < 0.001). Soil pH and these three cations increased with increasing latitude (**Table 1**). Soil total C (TC) was significantly positively correlated with soil total N (TN) (*r* = 0.733, *P* < 0.001), and they both were correlated with soil pH and these three cations. The latitudes of sampling sites were not significantly correlated with TC, TN, soil 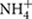 or 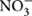 contents. Soil 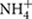 and 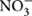 contents were significantly correlated with soil pH and TC, TN, exchangeable Ca^2+^ and exchangeable Mg^2+^ contents. Soil MBC had no significant relationship with other soil and site characteristics except for soil 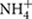 (**Table 1**).

**Table 1:** The correlation matrix between soil/site properties.

		Latitude	MAT	MAP	pH	ENa	EK	ECa	EMg	TC	TN	TP	AP	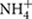	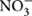
MAT	*r*	**-0.982**													
	*P*	0.000													
MAP	*r*	**-0.873**	**0.846**												
	*P*	0.000	0.000												
pH	*r*	**0.418**	**-0.434**	**-0.593**											
	*P*	0.000	0.000	0.000											
ENa	*r*	****-0.122	0.177	****-0.010	0.030										
	*P*	0.193	0.058	0.915	0.753										
EK	*r*	**0.333**	**-0.359**	**-0.476**	**0.685**	0.112									
	*P*	0.000	0.000	0.000	0.000	0.235									
ECa	*r*	**0.465**	**-0.484**	**-0.560**	**0.843**	****-0.023	**0.668**								
	*P*	0.000	0.000	0.000	0.000	0.807	0.000								
EMg	*r*	**0.506**	**-0.524**	**-0.569**	**0.776**	****-0.115	**0.610**	**0.886**							
	*P*	0.000	0.000	0.000	0.000	0.222	0.000	0.000							
TC	*r*	0.092	****-0.125	****-0.120	**0.358**	****-0.082	**0.331**	**0.651**	**0.612**						
	*P*	0.330	0.181	0.200	0.000	0.386	0.000	0.000	0.000						
TN	*r*	0.090	****-0.100	****-0.122	**0.382**	****-0.013	**0.311**	**0.575**	**0.605**	**0.733**					
	*P*	0.339	0.287	0.194	0.000	0.890	0.001	0.000	0.000	0.000					
TP	*r*	0.226	**-0.278**	****-0.173	**0.471**	****-0.216	**0.272**	**0.606**	**0.554**	**0.517**	**0.372**				
	*P*	0.015	0.003	0.065	0.000	0.021	0.003	0.000	0.000	0.000	0.000				
AP	*r*	**0.372**	**-0.412**	**-0.249**	0.234	**-0.284**	**0.326**	**0.310**	**0.345**	0.213	0.095	**0.638**			
	*P*	0.000	0.000	0.007	0.012	0.002	0.000	0.001	0.000	0.022	0.315	0.000			
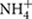	*r*	****-0.214	0.156	0.100	**0.267**	**-0.293**	0.175	**0.336**	**0.403**	**0.583**	**0.554**	0.219	****-0.032		
	*P*	0.021	0.097	0.289	0.004	0.001	0.062	0.000	0.000	0.000	0.000	0.019	0.733		
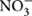	*r*	****-0.099	0.074	0.043	**0.500**	****-0.155	**0.291**	**0.603**	**0.638**	**0.709**	**0.628**	**0.568**	0.204	**0.560**	
	*P*	0.295	0.434	0.647	0.000	0.098	0.002	0.000	0.000	0.000	0.000	0.000	0.028	0.000	
MBC	*r*	****-0.121	0.104	0.089	****-0.169	****-0.114	0.002	-0.070	****-0.085	0.218	0.139	****-0.096	****-0.144	**0.269**	0.041
	*P*	0.197	0.269	0.345	0.072	0.226	0.982	0.456	0.366	0.019	0.139	0.309	0.124	0.004	0.660


**r* and *P* represent the Pearson’s correlation coefficient and the significance value, respectively. Values in bold indicate significant correlations (*P* < 0.01). MAT and MAP represent mean annual temperature and mean annual precipitation; EK, ENa, ECa and EMg represent soil exchangeable K^+^, Na^+^, Ca^2+^, and Mg^2+^ contents, respectively; TC, TN, TP, and AP represent soil total carbon, total nitrogen, total phosphorus and available phosphorus contents, respectively; 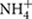 and 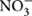 represent soil extractable 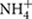 and 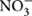 levels; MBC represents soil microbial biomass carbon.*

### Distribution and Abundance of Soil Bacterial Taxa

We obtained 4,667,656 sequences from all 115 samples, with an average of 40,588 sequences per sample. The range of sequences per sample in the whole dataset was from 7317 to 190250, and most samples (80%) had sequences between 16000 and 65000 (12 samples had less than 16000 sequences and 11 samples had more than 65000 sequences). The length of these sequences ranged of 191–351 bp, with a mean of 253 bp. Among these sequences, 98.6% could be classified. At the 97% similarity level, the sequences in all soils could be grouped into 325,433 phylotypes, with an average of 2,830 phylotypes per sample. *Actinobacteria, Acidobacteria, Alphaproteobacteria, Verrucomicrobia* and *Planctomycetes* (relative abundance >5%) were dominant groups across all sequence data, and they accounted for more than 73% of the bacterial sequences (**Supplementary Table S2**). Moreover, groups of *Chloroflexi, Betaproteobacteria, Deltaproteobacteria, Gammaproteobacteria, Gemmatimonadetes, Nitrospirae, Bacteroidetes* and *AD3* (relative abundance >1%) were less abundant (accounting for 22% of the bacterial sequences), but still existed in all soils. The rest of sequences could be classified into 56 groups, and 40 groups were rare (relative abundance <0.01%) (**Supplementary Table S2**).

### Dominant Bacterial Groups and Their Relationships with Soil/Site Properties

The abundance of some dominant bacterial groups was significantly correlated with soil and site characteristics (**Table 2**). The abundance of *Verrucomicrobia* (*r* = 0.472, *P* < 0.001; *r* = 0.511, *P* < 0.001), *Gemmatimonadetes* (*r* = 0.628, *P* < 0.001; *r* = 0.328, *P* < 0.001) and *Armatimonadetes* (*r* = 0.409, *P* < 0.001; *r* = 0.464, *P* < 0.001) increased with increasing geographic latitude; while the abundance of *Alphaproteobacteria* (*r* = -0.429, *P* < 0.001; *r* = -0.331, *P* < 0.001) and *Gammaproteobacteria* (*r* = -0.601, *P* < 0.001; *r* = -0.523, *P* < 0.001) decreased with increasing geographic latitude (**Table 2**). These five bacterial groups were also closely related to MAT and MAP of their locations (**Table 2**).

**Table 2:** The linear regression between the relative abundance of dominant bacterial groups (>0.1%) and their soil/site properties.

		Latitude	MAT	MAP	pH	ENa	EK	ECa	EMg	TC	TN	TP	AP	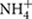	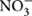	MBC
*Actinobacteria*	*r*	-0.135	**0.243**	0.130	-0.172	0.051	-0.194	**-0.243**	**-0.265**	**-0.365**	**-0.269**	-0.185	-0.109	**-0.355**	-0.218	-0.147
	*P*	0.152	0.009	0.166	0.066	0.588	0.038	0.009	0.004	0.000	0.004	0.048	0.245	0.000	0.019	0.117
*Acidobacteria*	*r*	0.124	-0.180	-0.157	0.115	-0.017	0.214	0.146	0.194	0.219	0.190	0.003	0.097	0.165	0.107	-0.019
	*P*	0.186	0.054	0.095	0.220	0.858	0.022	0.120	0.038	0.019	0.042	0.974	0.304	0.078	0.256	0.838
*Alphaproteobacteria*	*r*	**-0.429**	**0.403**	**0.362**	**-0.287**	0.028	**-0.293**	**-0.323**	**-0.403**	-0.181	-0.130	**-0.243**	**-0.249**	0.070	-0.169	0.015
	*P*	0.000	0.000	0.000	0.002	0.765	0.002	0.000	0.000	0.053	0.165	0.009	0.007	0.457	0.071	0.878
*Verrucomicrobia*	*r*	**0.472**	**-0.499**	**-0.394**	-0.053	0.022	-0.036	-0.046	0.147	-0.064	-0.092	0.052	0.171	-0.175	-0.228	0.045
	*P*	0.000	0.000	0.000	0.575	0.811	0.706	0.623	0.118	0.495	0.331	0.578	0.067	0.061	0.014	0.630
*Planctomycetes*	*r*	-0.098	0.064	0.100	-0.187	-0.027	-0.039	-0.105	-0.037	0.190	0.114	-0.029	0.023	0.182	0.121	0.109
	*P*	0.298	0.496	0.286	0.045	0.774	0.681	0.263	0.696	0.042	0.223	0.761	0.811	0.051	0.198	0.248
*Chloroflexi*	*r*	0.233	-0.204	-0.212	**0.363**	0.014	**0.383**	**0.401**	**0.355**	0.149	0.132	0.200	0.158	-0.061	**0.252**	0.094
	*P*	0.012	0.029	0.023	0.000	0.881	0.000	0.000	0.000	0.112	0.158	0.032	0.091	0.518	0.007	0.320
*Betaproteobacteria*	*r*	-0.060	-0.013	0.065	**0.343**	-0.200	**0.296**	**0.304**	0.208	0.146	0.156	0.206	0.005	**0.316**	0.213	-0.036
	*P*	0.524	0.892	0.492	0.000	0.032	0.001	0.001	0.026	0.119	0.096	0.027	0.957	0.001	0.022	0.706
*Deltaproteobacteria*	*r*	-0.121	0.095	-0.029	**0.408**	0.087	**0.246**	**0.407**	0.222	**0.294**	**0.268**	0.153	-0.175	**0.320**	**0.276**	0.047
	*P*	0.199	0.314	0.759	0.000	0.357	0.008	0.000	0.017	0.001	0.004	0.101	0.062	0.000	0.003	0.617
*Gammaproteobacteria*	*r*	**-0.601**	**0.581**	**0.560**	-0.130	-0.003	-0.161	-0.046	-0.145	**0.265**	0.191	0.159	-0.092	**0.393**	**0.363**	0.126
	*P*	0.000	0.000	0.000	0.166	0.972	0.085	0.628	0.122	0.004	0.040	0.089	0.326	0.000	0.000	0.181
*Gemmatimonadetes*	*r*	**0.628**	**-0.597**	**-0.649**	**0.506**	0.006	**0.394**	**0.442**	**0.360**	-0.058	0.028	0.062	0.103	-0.237	-0.081	**-0.241**
	*P*	0.000	0.000	0.000	0.000	0.952	0.000	0.000	0.000	0.541	0.763	0.514	0.274	0.011	0.389	0.009
*Nitrospirae*	*r*	0.087	-0.155	-0.168	**0.540**	-0.038	**0.330**	**0.521**	**0.410**	**0.314**	**0.282**	**0.307**	0.009	**0.318**	**0.303**	-0.037
	*P*	0.357	0.099	0.073	0.000	0.687	0.000	0.000	0.000	0.001	0.002	0.001	0.927	0.001	0.001	0.693
*Bacteroidetes*	*r*	0.134	-0.157	-0.167	**0.517**	-0.222	**0.368**	**0.611**	**0.589**	**0.567**	**0.494**	**0.525**	**0.297**	**0.575**	**0.646**	0.057
	*P*	0.154	0.093	0.075	0.000	0.017	0.000	0.000	0.000	0.000	0.000	0.000	0.001	0.000	0.000	0.547
*Firmicutes*	*r*	-0.131	0.167	0.030	0.011	0.124	-0.154	-0.209	-0.201	**-0.270**	-0.235	-0.184	0.042	-0.131	-0.118	-0.095
	*P*	0.164	0.075	0.749	0.911	0.187	0.100	0.025	0.031	0.004	0.011	0.049	0.659	0.163	0.209	0.312
*Armatimonadetes*	*r*	**0.409**	**-0.375**	**-0.345**	0.138	-0.019	0.020	0.209	0.165	0.039	0.083	-0.004	-0.034	-0.114	-0.059	0.053
	*P*	0.000	0.000	0.000	0.141	0.844	0.831	0.025	0.078	0.680	0.376	0.964	0.721	0.223	0.528	0.572


**r* and *P* represent the correlation coefficient of the linear regression and the significance value, respectively. Values in bold indicate significant correlations (*P* < 0.01). MAT and MAP represent mean annual temperature and mean annual precipitation; EK, ENa, ECa and EMg represent soil exchangeable K^+^, Na^+^, Ca^2+^, and Mg^2+^ contents, respectively; TC, TN, TP, and AP represent soil total carbon, total nitrogen, total phosphorus and available phosphorus contents, respectively; 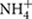 and 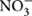 represent soil extractable 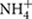 and 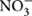 levels; MBC represents soil microbial biomass carbon.*

**FIGURE 2:**
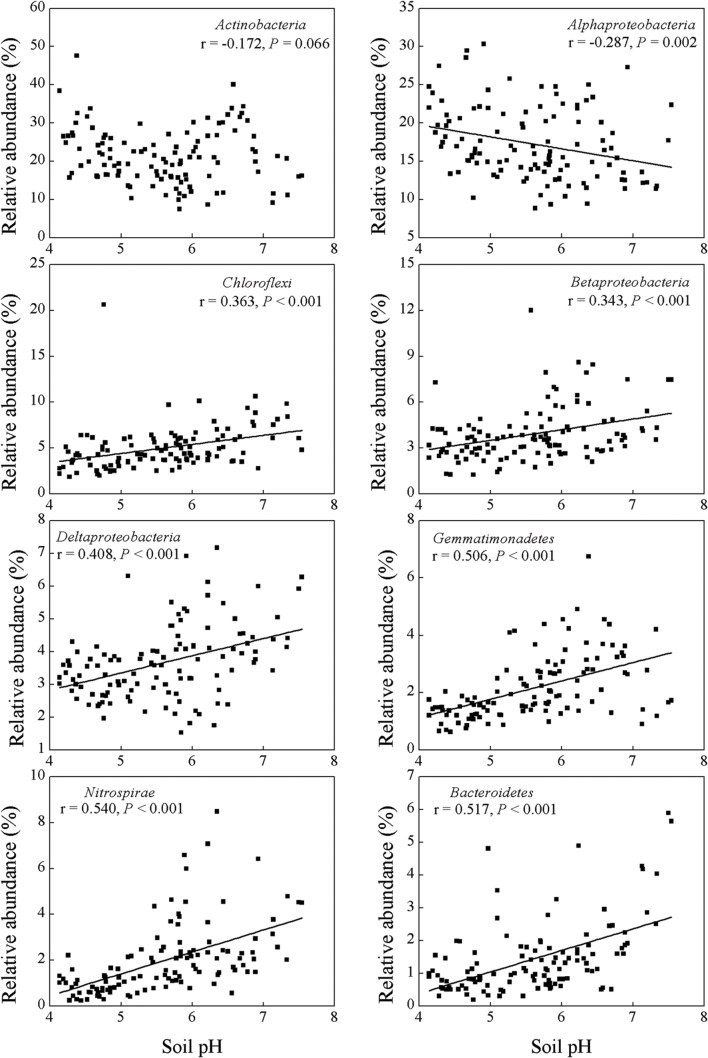
**Relationships between the relative abundance of dominant bacterial groups and soil pH**.

**FIGURE 3:**
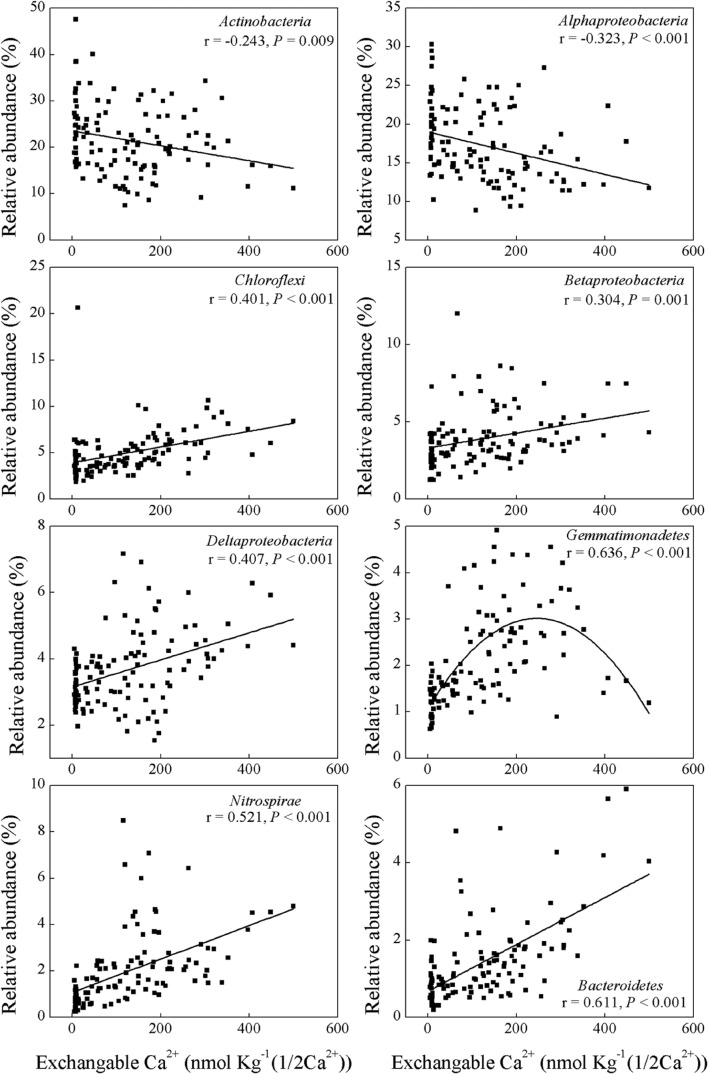
**Relationships between the relative abundance of dominant bacterial groups and soil exchangeable Ca^2+^ content**.

Soil pH and exchangeable K^+^, Ca^2+^, and Mg^2+^ contents affected many bacterial groups. For example, the relative abundance of *Chloroflexi, Betaproteobacteria, Deltaproteobacteria, Gemmatimonadetes, Nitrospirae* and *Bacteroidetes* was positively correlated with soil pH and exchangeable K^+^, Ca^2+^, and Mg^2+^ contents, while the relative abundance of *Alphaproteobacteria* was negatively correlated with these parameters (**Table 2**; **Figures 2** and **3**). Although the *Acidobacteria* group did not show correlation with soil pH and exchangeable K^+^, Ca^2+^, and Mg^2+^ contents, most of *Acidobacteria* subgroups had significant correlations with these parameters (**Supplementary Table S3**; **Supplementary Figures S1** and **S2**).

Soil total carbon, nitrogen and phosphorus were important factors for some bacterial groups. For example, these three parameters were all positively correlated with the relative abundance of *Nitrospirae* and *Bacteroidetes* (**Table 2**; **Supplementary Figures S3** and **S4**). The relative abundance of *Deltaproteobacteria* and *Gammaproteobacteria* showed positive correlations with TC and TN, while the abundance of *Actinobacteria* and *Firmicutes* showed negative correlations with TC and TN. In addition, the relative abundance of *Alphaproteobacteria* was only correlated with TP but not with TC and TN.

Soil available phosphorus (AP) and extractable 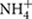 and 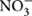 contents are nutrients directly used by microbes and therefore were related to many bacterial groups. Soil AP was positively correlated with *Bacteroidetes* but negatively correlated with *Alphaproteobacteria*. The relative abundance of *Actinobacterial, Betaproteobacteria, Deltaproteobacteria, Gammaproteobacteria, Nitrospirae* and *Bacteroidetes* had significantly positive relationship with soil extractable 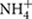 and 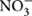 contents. Soil MBC was only correlated with the relative abundance of *Gemmatimonadetes* group.

### Soil Bacterial Community Diversity

**FIGURE 4:**
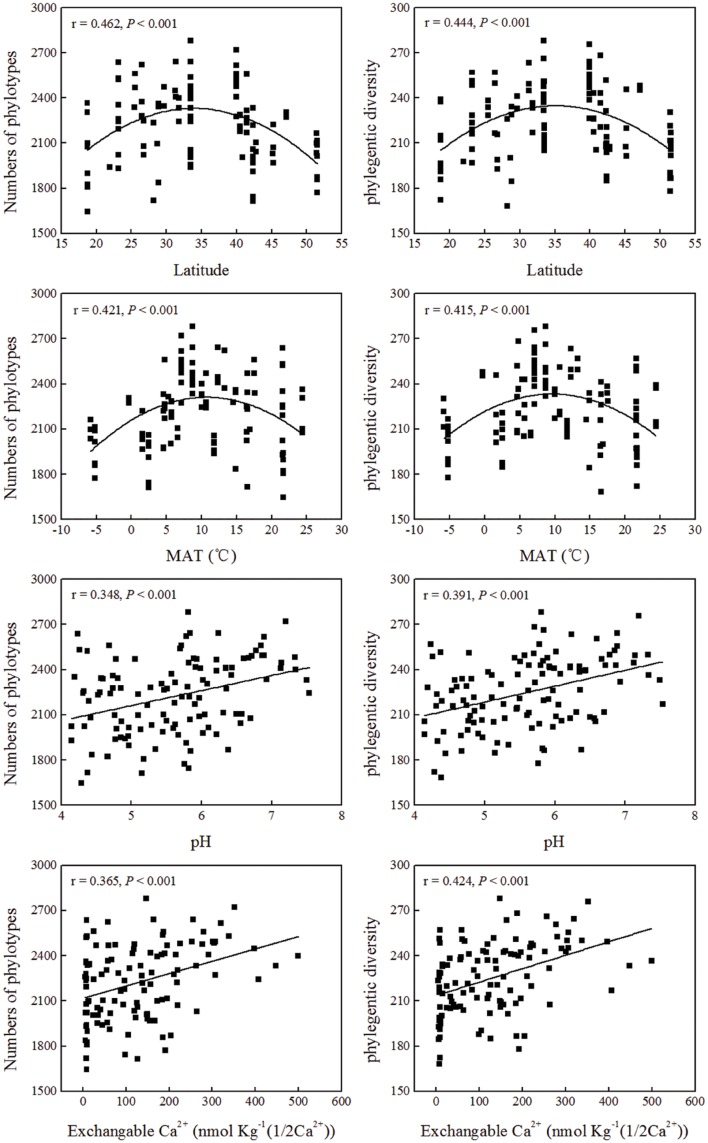
**Relationship between soil bacterial diversity indices (phylotypes and phylogenetic diversity) and soil location, or soil physical and chemical properties**.

The pairwise correlation analysis between the indices of the soil bacterial community diversity and soil location, or soil physical and chemical properties showed that soil pH, TC and exchangeable K^+^, Ca^2+^, and Mg^2+^ and extractable 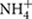 and 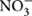 contents were significantly positively correlated with both phylotype richness and phylogenetic diversity (**Table 3**; **Figure 4**). Soil TN was positively correlated with phylogenetic diversity. A parabolic relationship was found between MAT and bacterial phylotype richness, and between MAT and phylogenetic diversity (**Figure 4**). Latitude presented similar effects on these indices of the soil bacterial community diversity with the tipping point at between 33.5°N and 40°N.

**Table 3:** The pairwise correlation analysis between soil bacterial diversity indices (phylotypes and phylogenetic diversity, PD for short) and soil location, physical and chemical properties.

		Latitude	MAT	MAP	pH	ENa	EK	ECa	EMg	TC	TN	TP	AP	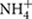	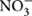	MBC
Phylotypes	*r*	-0.112	0.115	0.038	**0.348**	-0.007	**0.391**	**0.365**	0.210	0.213	0.161	0.096	-0.095	**0.261**	**0.260**	0.020
	*P*	0.232	0.220	0.684	0.000	0.939	0.000	0.000	0.024	0.022	0.086	0.305	0.314	0.005	0.005	0.836
PD	*r*	-0.008	0.014	-0.071	**0.391**	0.025	**0.422**	**0.424**	**0.301**	**0.255**	0.226	0.070	-0.105	**0.261**	**0.260**	0.029
	*P*	0.929	0.884	0.448	0.000	0.791	0.000	0.000	0.001	0.006	0.015	0.454	0.262	0.005	0.005	0.759


*Phylotypes were indentified at the 97% sequence similarity level. *r* and *P* represent the coefficient of the linear regression and the significance value, respectively. Values in bold indicate significant correlations with *P* < 0.01. MAT and MAP represent mean annual temperature and mean annual precipitation; EK, ENa, ECa, and EMg represent soil exchangeable K^+^, Na^+^, Ca^2+^, and Mg^2+^ contents, respectively; TC, TN, TP, and AP represent soil total carbon, total nitrogen, total phosphorus and available phosphorus contents, respectively; 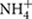 and 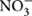 represent soil extractable 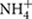 and 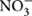 levels; MBC represents soil microbial biomass carbon.*

### Soil Bacterial Community Composition

**FIGURE 5:**
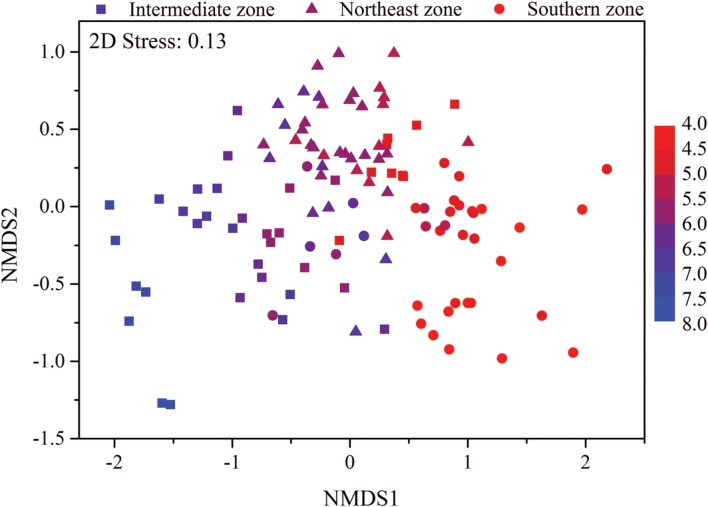
**Non-metric multidimensional scaling (NMDS) plot of bacterial community composition from 115 sites in typical Chinese forest soils.** Sites are color coded according to the soil pH values. The filled circle represent sites in southern zone with latitude between 18.70°N and 29.65°N, the filled triangles represent sites in northeast zone with latitude between 40.51°N and 51.53°N and the filled squares represent sites in intermediate zone with latitude between 31.30°N and 39.96°N.

**FIGURE 6:**
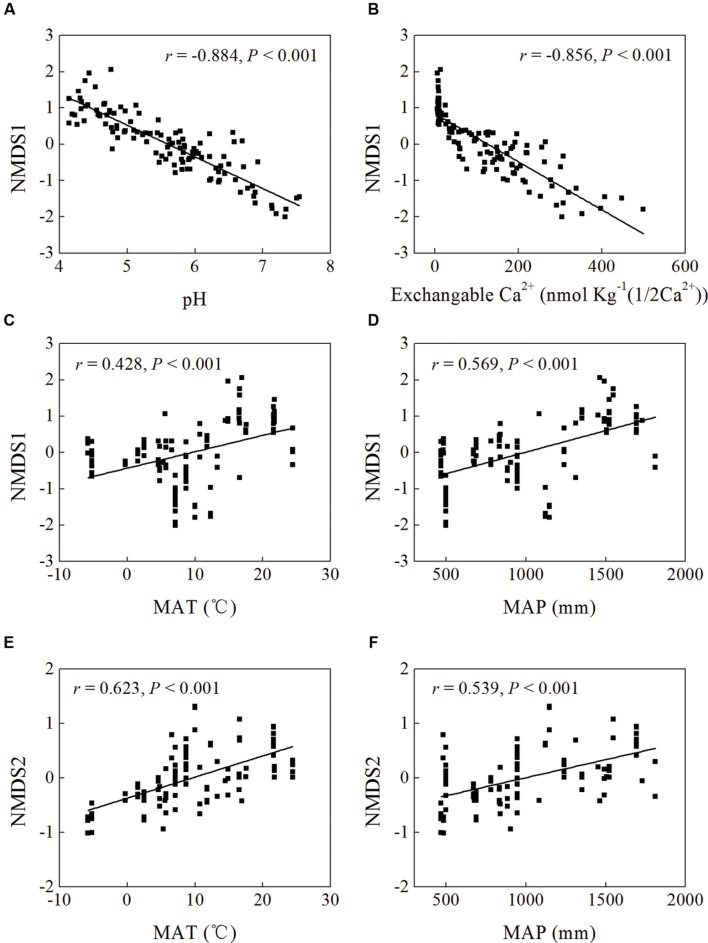
**Linear regression between non-metric multidimensional scaling (NMDS) scores (NMDS1 or NMDS2) and soil pH **(A)**, exchangeable Ca^2+^ content **(B)**, and MAT **(C,E)** and MAP **(D,F)** of sampling sites**.

Mantel test showed that “Bray–Curtis” distances of bacterial community composition was positively correlated with geographic distances (*r* = 0.259, *P* < 0.001, **Table 4**). Except for soil available phosphorus and MBC, all other examined environmental variables presented significant correlations with soil microbial community composition (**Table 4**). Soil pH was most strongly correlated with bacterial community composition (*r* = 0.700, *P* < 0.001). Utilization of both soil pH and soil TN predicted bacterial community composition better (*r* = 0.708, *P* < 0.001), while the addition of the other factors did not improve the regression’s efficiency. Moreover, NMDS visualization showed that variation in bacterial community composition was associated with variation in soil pH and differences in geographic region (**Figure 5**). Similarly, the significantly linear relationship between NMDS1 of NMDS scores and soil pH confirmed the importance of soil pH (**Figure 6A**). Soil exchangeable Ca^2+^ content had similar effects on soil bacterial community composition to soil pH (**Figure 6B**). Additionally, both NMDS1 and NMDS2 scores were closely correlated with MAT and MAP (**Figures 6C–F**). CCA analysis showed that soil pH, exchangeable Ca^2+^ and Mg^2+^, soil 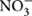, and MAT and MAP of soil locations were important factors on soil bacterial community composition (**Supplementary Figure S6**). The directions of pH, exchangeable Ca^2+^ and Mg^2+^ were closely correlated with CCA1, while the directions of soil 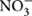, MAT and MAP were correlated with both CCA1 and CCA2.

**Table 4:** Correlations between “Bray–Curtis” distance of bacterial community composition and geographic or environmental variables distance using Mantel test.

Variable	*r*	*P*
Geographic distance	0.259	0.001
pH	0.700	0.001
ECa	0.626	0.001
EMg	0.535	0.001
EK	0.394	0.001
ENa	0.151	0.003
MAT	0.257	0.001
MAP	0.415	0.001
TC	0.317	0.001
TN	0.344	0.001
TP	0.268	0.001
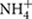	0.233	0.001
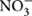	0.392	0.001
AP	0.081	0.131
MBC	0.055	0.240
pH+ECa	0.626	0.001
pH+MAT	0.270	0.001
pH+MAP	0.415	0.001
pH+TC	0.430	0.001
pH+TN	0.708	0.001
pH+TP	0.268	0.001
pH+ 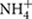	0.240	0.001
pH+ 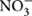	0.398	0.001
pH+ECa+EMg+EK	0.628	0.001
pH+MAT+MAP	0.415	0.001
pH+TC+TN	0.431	0.001
pH+ 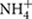 + 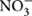	0.358	0.001
pH+ECa+MAP+TN+ 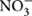	0.475	0.001


**r* and *P* represent the correlation coefficient and the significance value, respectively. MAT and MAP represent mean annual temperature and mean annual precipitation; EK, ENa, ECa, and EMg represent soil exchangeable K^+^, Na^+^, Ca^2+^, and Mg^2+^ contents, respectively; TC, TN, TP, and AP represent soil total carbon, total nitrogen, total phosphorus and available phosphorus contents, respectively; 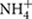 and 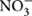 represent soil extractable 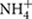 and 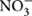 levels; MBC represents soil microbial biomass carbon.*

**FIGURE 7:**
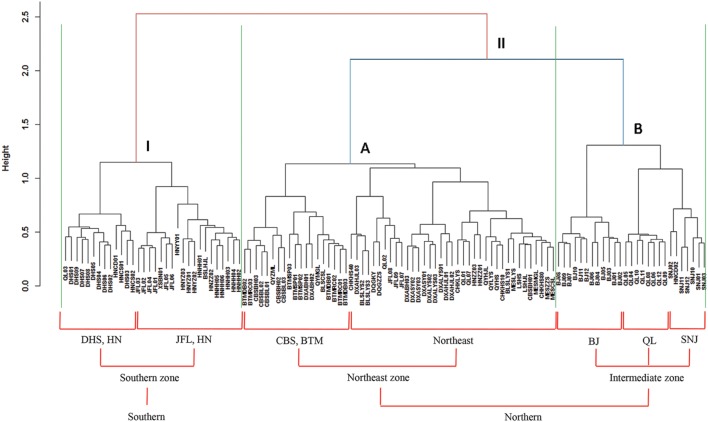
**Cluster analysis of bacterial communities based on “Bray–Curtis” dissimilarity matrix.** The symbols located at the ends of the cluster branch represented all the 115 soil samples and their detailed information are listed in **Supplementary Table S1**. All the 115 soil samples were clustered into two main groups (I and II) which were roughly corresponding to southern and northern forest soils, respectively. Moreover, the group II was coarsely divided into two subgroups (A and B) which were mainly from northeast zone forest soils and intermediate zone forest soils, respectively.

**FIGURE 8:**
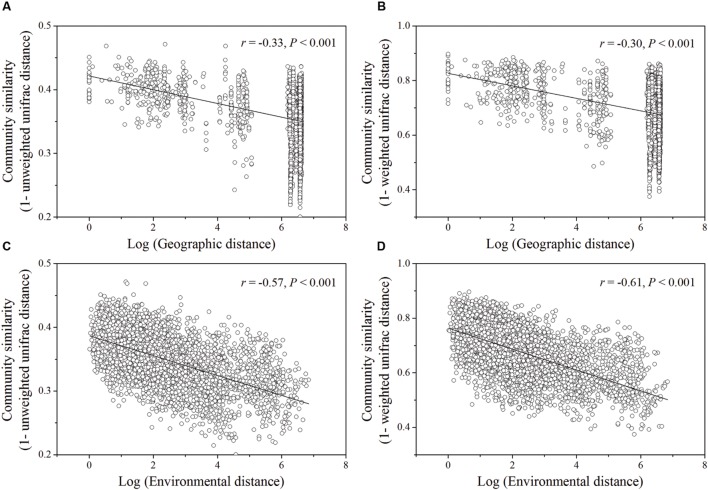
**Relationships between log of geographic distance **(A,B)** or environmental distance **(C,D)** and bacterial community similarity.** The significant vectors formed during PCNM analysis of geographic coordinates of sampling sties were used to construct geographic distance matrix. The environmental variables (pH, ECa, 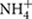) used to estimate environmental distance were selected by BioEnv procedure. Bacterial community similarity was estimated by pairwise Unifrac distance (unweighted or weighted) in community composition.

Based on the “Bray–Curtis” dissimilarity matrix, the bacterial communities of the 115 soils were roughly clustered into two big groups (**Figure 7**). Group I consisted of 32 samples which were mainly from southern forests of China with low latitudes (ranging from 18.70°N to 29.65°N). Group II was composed of 83 soils which were located in forests of northern China with middle latitudes ranging from 31.30°N to 51.53°N. Group II could be further divided into two subgroups (A and B). Subgroup A was consisted of 56 soils, most of which were sampled from northeastern China. Subgroup B contained 27 soils, which were sampled from Beijing, Qinling and Shennongjia with the latitude ranging from 31.30°N to 39.96°N (intermediate zone). These results corresponded to the results of NMDS analysis (**Figure 5**), which showed that bacterial community structure differed greatly between forest soils with acidic pH at lower latitude sites (less than 30°N) and forest soils with near-neutral, neutral or weakly alkaline pH at mid latitude (31°N – 40 °N) and high latitude sites (41°N – 52°N) in China. Additionally, the relationship between geographic distances, environmental distance and bacterial community similarity in community composition (**Figure 8**) indicated that more distinct bacterial communities could be found in two soils far from each other than in two soils with a near distance and also in two soils with more different soil properties.

**FIGURE 9:**
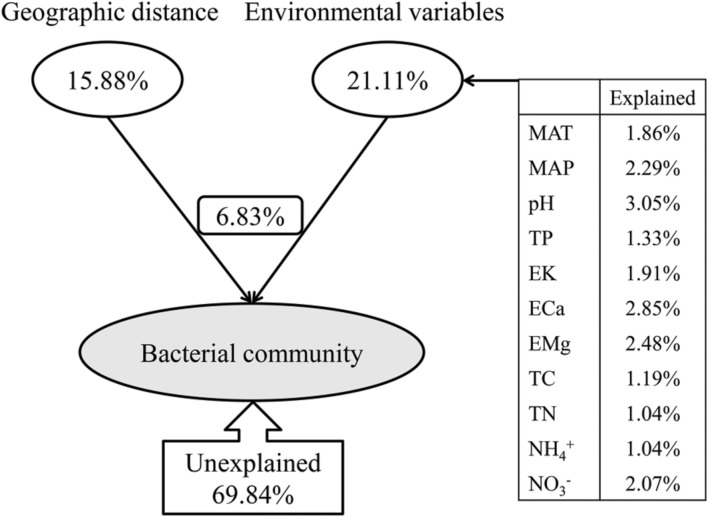
**Variation partition analysis of the effects of geographic distance and environmental variables on the bacterial community.** The environmental variables show different contributions to the variation of the bacteria community. MAT and MAP represent mean annual temperature and mean annual precipitation; TP represent soil total phosphorus contents; EK, ECa and EMg represent soil exchangeable K^+^, Ca^2+^ and Mg^2+^ contents, respectively; 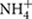 and 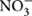 represent soil extractable 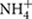 and 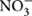 levels; MBC represents soil microbial biomass carbon.

The variance partitioning analysis showed the relative contributions of the geographic distance and environmental variables to the bacterial community structure (**Figure 9**). CCA analysis selected a subset of environmental variables (MAT, MAP, pH, TP, EK, ECa, EMg, TC, TN, 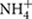 and 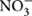) which together explained 21.11% of the bacterial community variation, more than the geographic distance (15.88%) (**Figure 9**). Therefore, the soil characteristics and environmental factors were more important than the geographic dispersal limitation in determining the bacterial community structure in Chinese forest soils. These selected environmental variables, i.e., MAT, MAP, pH, TP, EK, ECa, EMg, TC, TN, 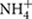 and 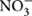, explained 1.86, 2.29, 3.05, 1.33, 1.91, 2.85, 2.48, 1.19, 1.04, 1.04, and 2.07% of the bacterial community variation, respectively. These environmental variables combining geographic distance explained 30.16% of the bacterial community variation, leaving 69.84% of unexplained variation, indicating that the overlapping effect of environmental variables and geographic distance on the bacterial community variation was 6.83% (**Figure 9**), and that there were many unmeasured or unknown factors that contributed to the large portion of unexplained variation in this study.

## Discussion

### Abundance of Dominant Bacterial Communities and Its Relationship with Climatic and Soil Factors

We found *Actinobacteria* phylum was the most dominant group (22%) in our studied forest soils. *Acidobacteria* accounted for 18% of all bacterial communities, while the relative abundance of *Bacteroidetes* was only about 1.4%. These results only partly agree with findings in the 88 soils across North and South America (Lauber et al., 2009) and the 26 black soils in northeastern China (Liu et al., 2014). They both found *Acidobacteria* was the most abundant phylum and *Bacteroidetes* was 11.2 and 5.6% as reported by Lauber et al. (2009) and Liu et al. (2014), respectively. Moreover, the relative abundance of *Verrucomicrobia* (8.68%) and *Planctomycetes* (6.75%) phyla in our study was much higher than results in Lauber et al. (2009) (0.9 and 0.09%, respectively) and Liu et al. (2014) (3.22 and 4.85%, respectively). Previous studies may have underestimated the abundance of *Verrucomicrobia* due to the bias of primers (Bergmann et al., 2011). However, using the same primers as this study, Fierer et al. (2012) found a huge variability in the relative abundance of the major bacterial taxa among different biomes including tropical forest, temperate forest, and boreal forest soils collected from different sites (for example, 5.22–40.29% for *Verrucomicrobia* and 1.61–5.56% for *Bacteroidetes*). Our results agreed with their findings and suggested that at large spatial scales, the dominant bacterial groups may be quite different among different regions.

We further studied the controlling factors of the dominant bacterial groups and found different bacterial groups responded differently to soil properties and local climate (MAT and MAP) gradients (**Table 2**). For example, *Alphaproteobacteria* and *Gammaproteobacteria* were more abundant in southern tropical zone than in northern temperate zone while *Verrucomicrobia*, *Gemmatimonadetes*, and *Armatimonadetes* presented the opposite trend. MAT and MAP may have played an important role in influencing some bacterial taxa, especially for *Verrucomicrobia* and *Armatimonadetes*, which had no or weak relevance to soil properties but strong relationship with local climate (**Table 2**). A significant negative correlation was observed between MAP and the relative abundance of *Verrucomicrobia* in forest soils in this study (**Table 2**), while the opposite trend was found in grassland soils of the arid and semiarid areas in China (Wang et al., 2015). Therefore, the responses of *Verrucomicrobia* to climatic conditions were different between arid/semiarid areas and semi-humid/humid areas, and that the intermediate amount of precipitation (e.g., 400–500 mm MAP) was probably the most beneficial for *Verrucomicrobia*. In general, the relative abundance of most phyla was positively correlated to soil parameters such as pH, exchangeable cations, C, N, P, 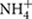 and 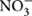 (**Table 2**; **Figure 2**), which indicated that most bacterial taxa exhibited copiotrophic attributes and seemed to be favored by neutral pH and high carbon availability. Prior studies also found that most bacteria benefits from optimum living conditions (McCaig et al., 1999; Axelrood et al., 2002; Padmanabhan et al., 2003; Fierer et al., 2007). However, *Actinobacteria*, *Alphaproteobacteria*, *Firmicutes* and the dominant subgroups of *Acidobacteria* (GP1 to GP3) showed negative relationships with those soil parameters (**Table 2**, **Supplementary Table S3**; **Figure 3**, **Supplementary Figures S1** and **S2**). Therefore, they possibly had oligotrophic lifestyle and were adapted to low-nutrient and low pH soils, which were consistent with previous reports (Fierer et al., 2007; Naether et al., 2012).

### Spatial Patterns of Bacterial Biogeographic Distribution

We found the relationship between soil bacterial diversity and latitude was a parabola shape with the tipping point (maximum) at between 33.50°N and 40°N (**Figure 4**), which falls into the warm-temperate zone of China with MAT of about 7–9°C (**Supplementary Table S1**; **Figure 4**). The changes of animal and plant diversity with latitudinal gradients have been well documented and studied for centuries with the well-established conclusion that plants and animals generally exhibit an increase of diversity with decreasing latitude (Lomolino et al., 2006). However, whether microbial diversity also exhibits a latitudinal gradient has not reached a general conclusion. Some studies found that bacterial diversity increased or decreased with latitude (Buckley et al., 2003; Fuhrman et al., 2008; Liu et al., 2014), while other studies found no relationship between bacterial diversity and latitude (Fierer and Jackson, 2006; Corby-Harris et al., 2007; Lauber et al., 2009; Chu et al., 2010). The latitudinal trend found in our study was based on the large scale data with a latitude range between 18.70°N and 51.53°N. The parabola shaped relationship between bacterial diversity and latitude indicated that optimum conditions for highest bacterial diversity in our studied areas were located at the warm-temperate zone and bacteria did not show a simply latitudinal diversity gradient as previously reported (Buckley et al., 2003; Fuhrman et al., 2008; Liu et al., 2014). In the areas with higher bacterial diversity, most forests belong to the deciduous broad-leaved forest type, which have higher substrate availability (such as C or N sources) for bacterial growth compared to coniferous forest (Huang et al., 2004; Geng et al., 2009), or have a more comfortable soil physical environment for bacteria (Wallenstein et al., 2007). The near-neutral pH (6–7) and temperate climate conditions (**Figure 4**) may also contribute to the high bacterial diversity in this area.

According to the dissimilarity matrix in the whole bacterial community composition, all 115 soil samples in this study were clustered into two main groups (**Figure 7**), which were located in the low latitude zone (18.70°N to 29.65°N) and the middle latitude zone (31.30°N to 52.53°N) (**Supplementary Table S1**). Moreover, a significant correlation was observed between geographic distances and bacterial community dissimilarities (**Figures 8A,B**). These findings suggested that the soil bacterial communities in the Chinese forest soils zone were distributed geographically. It is clear that soil bacterial communities in southern forests are distinct from those in northern forests and 30°N could be coarsely considered as the dividing line between them. However, some soil samples in tropical or subtropical forests (for examples JFL07, JFL08, JFL09, HNZZ01 and HNZZ03) were clustered into group A (mainly consist of northern temperate and boreal forest soils); while some bacterial community structure in temperate forest soils (BSLHJL and QL03) were more similar to that in southern forest soils (group I). This result suggested that soil properties and climatic factors (**Figures 6** and **8C,D**) also play a very important role in determining bacterial community composition.

### Determinant Factors of the Biogeographic Distribution of Soil Bacteria

The variation of soil bacterial phylotype and phylogenetic diversity along latitude gradients was similar to that along soil pH, ECa^2+^, EMg^2+^ and TC gradients (**Figure 4**; **Supplementary Figure S5**). Therefore, these soil properties and climatic conditions (MAT and MAP) together determined the biogeographic distribution of soil bacteria in our studied areas although these parameters were inter-correlated with each other (**Table 1**). Multiple variable analysis suggested that soil pH was the most important determinant of soil bacterial community structure (**Figures 5** and **6**; **Supplementary Figure S6**), which has been broadly documented in soils from a broad range of ecosystems (Fierer and Jackson, 2006; Baker et al., 2009; Liu et al., 2014). In fact, the cluster analysis results which divided bacterial composition into southern (Group I) and northern forest (Group II) groups (**Figure 7**) was also related to changes of soil pH because most soils (28 out of 32 samples) from Group I were acidic with pH < 5, while the soils classified into group II mainly (74 out of 83 samples) had pH > 5 (**Figure 5**, **Supplementary Table S1**). Our results (**Figure 4**) agreed with previous findings that acidic soils usually showed lower phylogenetic diversity than neutral soils (Fierer and Jackson, 2006; Lauber et al., 2009; Chu et al., 2010) and were mostly dominated by particular taxa (Griffiths et al., 2011). Therefore, soil pH = 5 probably can be used as a dividing line between northern and southern China regarding soil bacterial community composition and also a threshold below which soil bacterial diversity may decline and soil bacterial community structure may change significantly.

It is noteworthy that some soil exchangeable cations (Ca^2+^, Mg^2+^, K^+^) were also correlated with soil bacterial composition and diversity and some taxonomic groups (**Table 3**, **Figures 3** and **4**; **Supplementary Figure S2**). Soil pH strongly influenced these cations (**Table 1**), which agreed with previous findings conducted in tropical soils (Sanchez, 1977; Fearnside, 1984) and ferrosol soils (Lacey and Wilson, 2001). Therefore, it is understandable that the effects of these cations on microbial diversity were similar to the effects of soil pH. Likewise, the threshold of soil ECa^2+^ at about 32 mmol kg^-1^ can also be used for partitioning bacterial community structures into group I (31 in 32 samples with ECa^2+^s < 32 mmol kg^-1^) and group II (82 in 83 samples with ECa^2+^s > 32 mmol kg^-1^) (**Supplementary Table S1**), suggesting that soil ECa^2+^ may be another suitable marker for cluster-dividing of soil bacterial community composition besides soil pH.

We found environmental factors played a more important role in driving bacterial community pattern than geographic distance (explained 21.11 and 15.88% of the variation in bacterial community structure, respectively) at the large spatial scale of this study (**Figure 9**). This result was similar to the results obtained at a smaller scale in the black soils of northeast China (Liu et al., 2014), but different from a study conducted along a transect of arid and semi-arid grasslands in northern China, which showed geographic distance (36.02%) explained more of the variation in bacterial community structure than environmental variables (24.06%) (Wang et al., 2015). This was probably because the latter study was conducted along a latitudinal transect and the variations of climatic conditions and geographic distances were highly constrained. In addition, there was a significant correlation between geographic and environmental distance with a weak strength (‘Mantel test’, *r* = 0.16 for pearson’s rank correlation and *r* = 0.36 for spearman’s rank correlation, respectively, *p* = 0.001, data not shown) across sites in this study, and this indicates that variation in bacterial community composition may be associated with both geographic distance and environmental dissimilarity between sites.

## Conclusion

The soil bacterial phylogenetic diversity of typical eastern Chinese forests showed a parabola shape along latitude and the maximum diversity appeared at latitudes between 33.50°N and 40°N, an area characterized by warm-temperate zones and moderate temperature, neutral soil pH and high substrate availability (soil C and N) from dominant deciduous broad-leaved forests. The dissimilarity matrix results showed that the latitude of 30°N was the dividing line in bacterial community composition between southern and northern forests. Soil properties and climate conditions (MAT and MAP) greatly accounted for the differences in the soil bacterial structure among examined forests. Soil pH was the most important determinant while soil exchangeable cations, especially Ca^2+^, and some other soil variables also showed impacts on the composition and diversity of the soil bacterial community. Soil pH = 5 or ECa^2+^ = 32 mmol kg^-1^ may be used as indicators for differentiating southern acidic forest soils from northern temperate forest soils in bacterial community composition in China. The edaphic variables and environmental factors played a more important role than geographic dispersal limitation in determining the bacterial community structure in studied soils. This work for the first time identifies factors that govern the biogeography of bacteria in forest soils across China. Further research aimed at other non-forest soils are needed to comprehensively understand the biogeography of bacteria in soils from China.

## Author Contributions

Conceived and designed experiments: EB, QW, and ZX. Performed the experiments: ZX, QW, DG, PJ, and JW. Analyzed the data: ZX, EB, and JZ. Wrote the paper: ZX and EB. All authors read and approved the final manuscript.

## Conflict of Interest Statement

The authors declare that the research was conducted in the absence of any commercial or financial relationships that could be construed as a potential conflict of interest.

## Footnotes

1 http://www.worldclim.org/.

2 http://qiime.org/
