# Supplementary material for: Biogeographic Distribution Patterns of Bacteria in Typical Chinese Forest Soils
Source: Front Microbiol. 2016 Jul 13;7:1106. doi: 10.3389/fmicb.2016.01106 (PMC4942481; doi:10.3389/fmicb.2016.01106)
Supplement: Supplementary file 1 [file Table_1.DOC]

Table S1. Locations, forest types, and physical and chemical properties of the 115 soil samples in different forest types in China.

| Sample | Location | Latitude (N) and longitude (E) | Forest type | pH | Total C (%) | Total N  (%) | Total P  (mg kg^-1^) | Available P  (mg kg^-1^) | N-NH_4_^+^  (mg kg^-1^) | N-NO_3_^−^  (mg kg^-1^) | MBC  (mg kg^-1^)a | EK  (mmol kg^-1^)b | ENa  (mmol kg^-1^)b | ECa  (mmol kg^-1^ (1/2Ca^2+^))b | EMg  (mmol kg^-1^ (1/2Mg^2+^))b |
| --- | --- | --- | --- | --- | --- | --- | --- | --- | --- | --- | --- | --- | --- | --- | --- |
| JFL01 | Hainan, Jianfengling | 18.73° 108.87° | Tropical montane rain forest | 4.67 | 2.53 | 0.28 | 136.9 | 4.9 | 22.41 | 4.7 | 812.19 | 1.4 | 1.81 | 8.9 | 1.24 |
| JFL02 |  | 18.74° 108.84° |  | 4.28 | 8.51 | 0.61 | 152.96 | 5.36 | 18.2 | 10.05 | 1177.35 | 2.3 | 1.2 | 8.55 | 2.31 |
| JFL03 |  | 18.74° 108.86° |  | 4.66 | 1.79 | 0.14 | 119.3 | 3.46 | 15.02 | 6.91 | 578.11 | 1.84 | 1.31 | 9.71 | 1.2 |
| JFL04 |  | 18.72° 108.83° | Tropical evergreen monsoon forest | 4.97 | 1.36 | 0.11 | 117.57 | 3.01 | 22.79 | 5.51 | 462.09 | 2.84 | 2.04 | 10.26 | 2.57 |
| JFL05 |  | 18.72° 108.83° |  | 5.18 | 1.21 | 0.1 | 145.64 | 5.55 | 15.76 | 1.8 | 437.02 | 4.16 | 1.31 | 9.17 | 4.71 |
| JFL06 |  | 18.72° 108.83° |  | 5.46 | 1.13 | 0.11 | 131.44 | 2.14 | 18.51 | 2.6 | 341.99 | 5.88 | 1.42 | 14.16 | 7.64 |
| JFL07 |  | 18.70° 108.79° | Tropical semi-deciduous monsoon forest | 6.43 | 1.46 | 0.13 | 339.06 | 3.68 | 10.31 | 8.4 | 361.68 | 3.54 | 1.44 | 57.85 | 22.41 |
| JFL08 |  | 18.71° 108.79° |  | 6.08 | 1.34 | 0.13 | 726.42 | 20.03 | 12.51 | 11.85 | 403.87 | 4.09 | 1.77 | 55.93 | 18.25 |
| JFL09 |  | 18.71° 108.79° |  | 6.7 | 1.68 | 0.17 | 607.32 | 12.4 | 15.62 | 13.5 | 490.89 | 5.31 | 1.31 | 94.32 | 30.1 |
| XSBN01 | Yunnan, Xishuangbanna | 21.96° 101.20° | Tropical seasonal rain forest -100 years | 4.91 | 1.69 | 0.17 | 265.09 | 3.83 | 2.71 | 4.93 | 551.37 | 1.5 | 1.05 | 8.46 | 1.62 |
| DHS01 | Guangdong, Dinghushan | 23.17° 112.54° | Subtropical monsoon evergreen broad leaf forest | 4.14 | 2.9 | 0.22 | 239.88 | 3.76 | 9.87 | 4.2 | 414.48 | 1.61 | 1.16 | 7.92 | 1.07 |
| DHS02 |  | 23.17° 112.54° |  | 4.39 | 1.78 | 0.14 | 170.32 | 2.44 | 10.43 | 3.39 | 362.32 | 1.49 | 0.97 | 7.8 | 0.8 |
| DHS03 |  | 23.17° 112.54° |  | 4.14 | 3.47 | 0.35 | 164.31 | 3.58 | 16.04 | 4.63 | 387.61 | 1.36 | 1.03 | 9.16 | 1.04 |
| DHS04 |  | 23.17° 112.53° | Subtropical coniferous and broad-leaved mixed forest | 4.19 | 4.32 | 0.23 | 145.96 | 2.42 | 16.11 | 2.43 | 384.11 | 1.05 | 1.12 | 7.44 | 1.03 |
| DHS05 |  | 23.17° 112.53° |  | 4.31 | 2.42 | 0.17 | 122.3 | 1.15 | 12.25 | 2.31 | 453.18 | 1.45 | 1.5 | 7.48 | 0.78 |
| DHS06 |  | 23.17° 112.54° |  | 4.32 | 2.15 | 0.15 | 132.02 | 1.15 | 11.67 | 3.61 | 339.80 | 1.59 | 1.16 | 5.29 | 0.5 |
| DHS07 |  | 23.17° 112.54° | Subtropical Pinus massoniana forest | 4.26 | 2.16 | 0.14 | 190.16 | 5.42 | 4.47 | 3.22 | 347.30 | 1.26 | 0.92 | 9.67 | 1.13 |
| DHS08 |  | 23.17° 112.54° |  | 4.37 | 1.65 | 0.11 | 143.85 | 2.26 | 4.95 | 1.84 | 294.45 | 0.9 | 0.82 | 8.23 | 0.66 |
| DHS09 |  | 23.17° 112.54° |  | 4.23 | 1.96 | 0.13 | 186.83 | 2.87 | 6.41 | 2.25 | 275.23 | 0.97 | 1.08 | 8.46 | 0.69 |
| HNYZ01 | Hunan, Yongzhou | 25.49° 111.39° | Subtropical evergreen and deciduous broad-leaved mixed forest | 4.6 | 2.2 | 0.2 | 417.62 | 1.51 | 7.86 | 8.29 | 1261.13 | 3.49 | 0.99 | 10.66 | 2.54 |
| HNYZ02 |  | 25.51° 111.49° |  | 5.05 | 2.41 | 0.2 | 480.59 | 1.38 | 14.61 | 5.98 | 1107.66 | 5.47 | 1 | 31.43 | 4.88 |
| HNYZ03 |  | 25.53° 111.40° |  | 4.68 | 1.92 | 0.19 | 1219.93 | 25.94 | 15.66 | 10.25 | 249.80 | 4.07 | 1.1 | 25.79 | 3.07 |
| HNZZ01 | Hunan, Zhuzhou | 26.50° 114.06° | Subtropical evergreen broad-leaved forest | 5.78 | 1.27 | 0.12 | 676.83 | 9.57 | 10.6 | 13.91 | 251.47 | 5.29 | 0.86 | 58.71 | 7.17 |
| HNZZ02 |  | 26.50° 114.06° |  | 4.74 | 2.57 | 0.16 | 437.28 | 4.06 | 22.14 | 1.02 | 284.18 | 2.19 | 0.39 | 6.9 | 0.85 |
| HNZZ03 |  | 26.50° 114.06° |  | 5.57 | 2.31 | 0.18 | 845.04 | 8.57 | 13.04 | 15.19 | 556.06 | 5.86 | 0.51 | 66.79 | 17.66 |
| HNHH01 | Hunan, Huitong | 28.86° 110.45° | Subtropical coniferous and broad-leaved mixed forest | 4.44 | 5.4 | 0.3 | 293.15 | 2.86 | 25.88 | 1.24 | 852.91 | 1.65 | 1.53 | 5.52 | 1.12 |
| HNHH02 |  | 28.85° 110.45° |  | 4.81 | 4 | 0.31 | 343.34 | 6.64 | 26 | 12.13 | 1061.37 | 1.62 | 1.13 | 15.03 | 2.4 |
| HNHH03 |  | 28.86° 110.45° |  | 4.74 | 6.8 | 0.48 | 1212.47 | 18 | 33.87 | 2.92 | 1091.52 | 3.12 | 1.26 | 6.89 | 1.93 |
| HNHH04 | Hunan, Huaihua | 26.81° 109.88° | Subtropical coniferous-deciduous mixed forests | 4.55 | 3.03 | 0.25 | 368.04 | 7.8 | 20.39 | 1.6 | 869.07 | 2.07 | 1.28 | 7.95 | 1.57 |
| HNHH05 |  | 26.81° 109.88° | Subtropical coniferous and broad-leaved mixed forest | 4.33 | 3.09 | 0.19 | 153.32 | 4.04 | 16.04 | 2.21 | 620.36 | 2.13 | 0.91 | 6.57 | 1.2 |
| HNHH06 |  | 26.81° 109.88° |  | 4.42 | 1.71 | 0.13 | 105.44 | 2.58 | 13.96 | 2.11 | 1369.08 | 2.05 | 0.75 | 7.75 | 1.16 |
| HNCS01 | Hunan, Changsha | 28.18° 113.65° | Subtropical coniferous and broad-leaved mixed forest | 4.53 | 1.83 | 0.15 | 336.32 | 3.87 | 14.79 | 8.83 | 1366.32 | 2.03 | 1.38 | 7.41 | 1.45 |
| HNCS02 |  | 28.18° 113.65° |  | 4.38 | 2.98 | 0.18 | 363.1 | 6.28 | 18.16 | 2.25 | 704.98 | 1.7 | 1.06 | 7.87 | 1.63 |
| HNYY01 | Hunan, Yueyang | 28.67° 113.62° | Subtropical evergreen and deciduous broadleaved mixed forest | 4.76 | 0.8 | 0.09 | 188.77 | 0.74 | 2.92 | 1.94 | 1342.64 | 1.47 | 0.66 | 13.12 | 1.5 |
| HNCD01 | Hunan, Changde | 29.65° 111.30° | Subtropical coniferous and broad-leaved mixed forest | 4.57 | 1.87 | 0.15 | 215.05 | 5.4 | 15.63 | 10.07 | 1761.33 | 1.2 | 1 | 14.98 | 1.52 |
| HNCD02 |  | 29.64° 111.29° | Subtropical evergreen and deciduous broad-leaved mixed forest | 5.93 | 2.05 | 0.17 | 303.46 | 3.12 | 15.34 | 8.67 | 1020.72 | 2.26 | 1.31 | 75.6 | 7.67 |
| SNJ01 | Hubei, Huanglianba | 31.30° 110.48° | Subtropical evergreen and deciduous broad-leaved mixed forest | 7.14 | 13.86 | 1.01 | 1288.33 | 8.33 | 73.64 | 111.55 | 812.31 | 5.82 | 0.29 | 396.96 | 89.82 |
| SNJ02 |  | 31.31° 110.48° |  | 6.24 | 6.95 | 0.51 | 505.01 | 5.5 | 90.47 | 22.25 | 1354.94 | 6.35 | 0.48 | 163.8 | 41.89 |
| SNJ03 |  | 31.31° 110.48° |  | 7.13 | 3.77 | 1.05 | 860.91 | 9.15 | 82.92 | 47.54 | 611.39 | 5.76 | 0.49 | 291.18 | 66.78 |
| SNJ10 | Hubei,  Shennongjia | 31.82° 110.51° | Subtropical evergreen and deciduous broad-leaved mixed forest | 7.34 | 11.08 | 0.73 | 4100.11 | 87.15 | 52.06 | 65.33 | 288.19 | 7.4 | 0.58 | 499.06 | 64.73 |
| SNJ11 |  | 31.82° 110.51° |  | 7.5 | 9.31 | 0.62 | 5418.51 | 60.56 | 33.36 | 69.54 | 543.82 | 5.6 | 0.72 | 447.65 | 50.8 |
| SNJ12 |  | 31.82° 110.51° |  | 7.54 | 8.86 | 0.57 | 4503.51 | 67.07 | 49.55 | 49.34 | 352.15 | 5.71 | 0.49 | 406.68 | 50.22 |
| QL01 | Shanxi, Qinling | 33.44° 108.44° | Temperate Sharp tooth oak forest | 5.99 | 4.02 | 0.25 | 384.18 | 2.43 | 22.69 | 0.77 | 618.94 | 4.54 | 0.99 | 182.29 | 17.19 |
| QL02 |  | 33.44° 108.44° |  | 5.84 | 4.73 | 0.29 | 512.53 | 2.88 | 35.1 | 0.63 | 950.71 | 4.98 | 0.7 | 255.94 | 15.51 |
| QL03 |  | 33.44° 108.44° |  | 6.22 | 3.51 | 0.27 | 460.48 | 1.72 | 31.31 | 1.96 | 655.26 | 6.14 | 0.62 | 196.17 | 26.91 |
| QL04 |  | 33.44° 108.45° | Temperate Chinese pine forest | 5.92 | 2.39 | 0.19 | 485.63 | 1.42 | 20.23 | 3.53 | 416.99 | 5.2 | 1.05 | 156.59 | 21.37 |
| QL05 |  | 33.44° 108.45° |  | 6.93 | 3.39 | 0.17 | 329.83 | 2.14 | 25.04 | 0.52 | 545.89 | 5.98 | 1.12 | 262.22 | 18.74 |
| QL06 |  | 33.44° 108.45° |  | 6.44 | 3.04 | 0.24 | 438.31 | 1.88 | 29.08 | 4.13 | 622.84 | 8.11 | 1.14 | 189.99 | 20.1 |
| QL07 |  | 33.43° 108.44° | Temperate Armand pine and sharp tooth oak forest | 5.82 | 3.77 | 0.26 | 379.25 | 2.01 | 25.99 | 0.77 | 599.03 | 4.44 | 1.22 | 118.87 | 16.52 |
| QL08 |  | 33.43° 108.44° |  | 6.35 | 2.57 | 0.2 | 414.86 | 3.86 | 24.16 | 2.29 | 580.37 | 6.88 | 1.06 | 115.63 | 14.01 |
| QL09 |  | 33.44° 108.44° |  | 5.89 | 3.53 | 0.25 | 428.93 | 2.29 | 19.25 | 3.39 | 640.91 | 7 | 1.13 | 119.21 | 15.19 |
| QL10 |  | 33.43° 108.46° | Temperate Chinese pine and sharp tooth oak forest | 5.7 | 5.72 | 0.41 | 559.2 | 4.74 | 37.11 | 3.63 | 1096.08 | 6.36 | 1.41 | 185.3 | 22.42 |
| QL11 |  | 33.43° 108.45° |  | 5.81 | 3.92 | 0.31 | 468.56 | 3.87 | 34.46 | 6.38 | 721.83 | 7.22 | 0.86 | 147.7 | 20.91 |
| QL12 |  | 33.43° 108.46° |  | 6.22 | 3.74 | 0.32 | 961.2 | 5.58 | 25.29 | 12.42 | 1018.26 | 5.57 | 0.65 | 173.31 | 19.73 |
| BTMDB01 | Henan, Baotianman | 33.50° 111.93° | Temperate Quercus glandulifera forest | 4.86 | 2.94 | 0.2 | 231.66 | 2.33 | 34.36 | 2.91 | 715.34 | 2.24 | 0.69 | 33.4 | 9.65 |
| BTMDB02 |  | 33.50° 111.93° |  | 4.79 | 3.15 | 0.22 | 273.75 | 5.05 | 23.63 | 0.91 | 761.92 | 2.55 | 0.58 | 35.69 | 7.9 |
| BTMDB03 |  | 33.50° 111.93° |  | 4.85 | 2.39 | 0.17 | 204.82 | 1.86 | 15.13 | 1.2 | 588.42 | 1.82 | 0.69 | 33.87 | 8.9 |
| BTMRC01 |  | 33.50° 111.93° | Temperate Sharp tooth oak forest | 4.77 | 4.14 | 0.26 | 305.82 | 2.6 | 29.88 | 0.64 | 721.10 | 2.39 | 0.94 | 44.84 | 9.23 |
| BTMRC02 |  | 33.50° 111.93° |  | 4.97 | 3.91 | 0.27 | 321.49 | 4.23 | 35.56 | 1.1 | 762.14 | 3.5 | 1.18 | 63.89 | 13.28 |
| BTMRC03 |  | 33.50° 111.93° |  | 4.96 | 3.41 | 0.26 | 387.28 | 4.31 | 31.12 | 1.13 | 817.68 | 2.65 | 0.69 | 56.76 | 15.22 |
| BTMSP01 |  | 33.50° 111.92° | Temperate Quercus variabilis forest | 4.7 | 2.13 | 0.13 | 271.44 | 3.41 | 15.72 | 0.49 | 596.59 | 2.71 | 0.71 | 25.44 | 6.74 |
| BTMSP02 |  | 33.50° 111.92° |  | 4.85 | 2.71 | 0.18 | 380.33 | 5.13 | 18.6 | 1.02 | 666.76 | 2.68 | 0.7 | 44.05 | 9.67 |
| BTMSP03 |  | 33.50° 111.92° |  | 4.78 | 2.03 | 0.14 | 384.54 | 5.86 | 15.93 | 1.29 | 578.57 | 2.8 | 0.61 | 60.42 | 14.58 |
| BJ01 | Beijing | 39.96° 115.42° | Temperate secondary birch forest | 6.6 | 4.64 | 0.34 | 560.26 | 3.72 | 37.71 | 10.54 | 786.85 | 7.49 | 0.85 | 277.32 | 38.24 |
| BJ02 |  | 39.95° 115.43° |  | 6.72 | 4.61 | 0.34 | 608.25 | 3.74 | 23.54 | 9.9 | 689.28 | 8.31 | 1.06 | 300.91 | 37.94 |
| BJ03 |  | 39.95° 115.43° |  | 6.78 | 4.84 | 0.36 | 952.08 | 4.87 | 24.24 | 6.73 | 798.09 | 10.46 | 1.13 | 338.18 | 38.98 |
| BJ04 |  | 39.96° 115.43° | Temperate oak mixed forest | 6.36 | 6.28 | 0.46 | 422.59 | 1.57 | 20.22 | 9.14 | 362.17 | 3.3 | 1.42 | 210.84 | 25.86 |
| BJ05 |  | 39.96° 115.43° |  | 6.67 | 3.15 | 0.25 | 458.08 | 1.91 | 5.47 | 17.84 | 606.43 | 5.89 | 1.56 | 225.38 | 28.97 |
| BJ06 |  | 39.96° 115.43° |  | 6.35 | 3.28 | 0.27 | 338.52 | 3.14 | 6.26 | 9.33 | 559.80 | 4.93 | 1.65 | 215.94 | 26.52 |
| BJ07 |  | 39.96° 115.42° | Temperate oak forest | 6.89 | 3.52 | 0.29 | 689.95 | 3.33 | 11.85 | 15.97 | 830.89 | 8.17 | 1.33 | 320.04 | 38.5 |
| BJ08 |  | 39.96° 115.42° |  | 6.87 | 4.2 | 0.36 | 629.81 | 3.28 | 14.71 | 10.26 | 618.53 | 9.95 | 1.29 | 252.34 | 31.5 |
| BJ09 |  | 39.96° 115.42° |  | 6.85 | 4.23 | 0.34 | 660.62 | 4.38 | 16.41 | 14.49 | 577.58 | 8.79 | 1.5 | 280.26 | 32.9 |
| BJ10 |  | 39.96° 115.44° | Temperate Juglans mandshurica forest | 7.2 | 6.49 | 0.57 | 1157.09 | 6.2 | 8.54 | 23.82 | 68.63 | 12.03 | 3.04 | 351.71 | 42.04 |
| BJ11 |  | 39.96° 115.44° |  | 6.89 | 5.68 | 0.5 | 911.28 | 3.6 | 25.64 | 16.72 | 917.38 | 10.21 | 1.48 | 307.04 | 40.73 |
| BJ12 |  | 39.96° 115.44° |  | 7.33 | 5.16 | 0.47 | 1014.75 | 6.4 | 6.52 | 38.73 | 705.37 | 14.7 | 1.29 | 304.09 | 37.83 |
| CHKHS10 | Liaoning, Caohekou | 40.51° 123.53° | Temperate Korean pine plantation -10 years | 5.8 | 4.49 | 0.39 | 637.74 | 2.27 | 13.42 | 1.69 | 600.21 | 4.62 | 1.26 | 160.05 | 20.64 |
| CHKHS40 |  | 40.51° 123.54° | Temperate Korean pine plantation -40 years | 5.27 | 2.16 | 0.17 | 169.66 | 1.49 | 10.39 | 4.91 | 345.88 | 3.77 | 1.17 | 82.78 | 10.75 |
| CHKHS80 |  | 40.51° 123.54° | Temperate Korean pine plantation -80 years | 5.62 | 2.54 | 0.22 | 471.87 | 3.12 | 8.05 | 1.62 | 313.79 | 4.36 | 1.83 | 96.41 | 14.05 |
| CHKLYS |  | 40.53° 123.56° | Temperate larch plantation-80 years | 5.97 | 3.26 | 0.22 | 298.97 | 3.91 | 8.7 | 2.47 | 486.59 | 2.27 | 1.28 | 156.38 | 17.66 |
| BSLHJ | Liaoning, Baishila | 40.86° 124.83° | Temperate coniferous and broadleaved mixed forest | 5.13 | 2.94 | 0.19 | 323.74 | 4.47 | 7.12 | 10.45 | 283.03 | 2.17 | 1.2 | 24.26 | 1.83 |
| QYHS | Liaoning, Qingyuan | 41.51° 124.56° | Temperate Korean pine forest | 5.91 | 2.89 | 0.24 | 385.08 | 3.02 | 14.57 | 3.85 | 508.18 | 2.24 | 1.38 | 142.49 | 21.33 |
| QYLYS |  | 41.51° 124.56° | Temperate larch forest | 5.47 | 2.52 | 0.26 | 1102.06 | 12.34 | 10.92 | 5.5 | 312.61 | 2.01 | 1.26 | 137.66 | 18.58 |
| QYZML |  | 41.51° 124.55° | Temperate weed trees forest | 5.63 | 3.82 | 0.3 | 428.45 | 2.95 | 18.32 | 1.68 | 499.00 | 6.34 | 1.07 | 108.46 | 25.07 |
| QYMGL |  | 41.51° 124.56° | Temperate Mongolian oak forest | 5.1 | 3.29 | 0.24 | 317.26 | 2.55 | 15.64 | 1.24 | 426.02 | 3.39 | 1.3 | 96.48 | 21.5 |
| QYHJL |  | 41.51° 124.56° | Temperate coniferous and broadleaved mixed forest | 5.71 | 4.65 | 0.46 | 1575.86 | 24.01 | 18.96 | 13.21 | 637.93 | 3.6 | 1.25 | 187.34 | 27.15 |
| BLSLYS1 | Liaoning, Binglashan | 42.35° 125.03° | Temperate larch plantation-30 years | 6.02 | 1.97 | 0.14 | 168.86 | 2.65 | 9.99 | 0.23 | 641.06 | 3.36 | 1.1 | 133.52 | 20.43 |
| BLSLYS2 |  | 42.35° 125.03° |  | 6.02 | 2.7 | 1.33 | 426.56 | 5.1 | 14.96 | 3.78 | 275.29 | 4.36 | 1.16 | 150.22 | 27.29 |
| BLSLYS3 |  | 42.35° 125.03° |  | 6.22 | 2.16 | 0.16 | 405.83 | 10.67 | 11.11 | 2.78 | 296.42 | 4.09 | 1.13 | 156.01 | 27.62 |
| BLSCSL |  | 42.35° 125.03° | Temperate secondary weed trees forest | 5.72 | 3.28 | 0.28 | 555.46 | 5.84 | 22.99 | 3.3 | 187.11 | 4.02 | 0.92 | 138.73 | 29.69 |
| CBSBL01 | Jilin, Changbaishan | 42.38° 128.08° | Temperate broad-leaved Korean pine forest | 5.78 | 3.5 | 0.32 | 567.6 | 3.53 | 17.1 | 7.35 | 1886.50 | 2.65 | 0.77 | 62.65 | 9.12 |
| CBSBL02 |  | 42.38° 128.08° |  | 5.45 | 3.81 | 0.31 | 482.27 | 8.87 | 16.48 | 5.47 | 798.74 | 5.24 | 1.48 | 122.32 | 26.02 |
| CBSBL03 |  | 42.38° 128.08° |  | 5.15 | 4.52 | 0.41 | 961.93 | 5.3 | 18.77 | 9.93 | 1003.00 | 4.22 | 1.72 | 126.92 | 32.95 |
| CBSBH01 |  | 42.39° 128.10° | Temperate birch forest | 5.6 | 4.23 | 0.35 | 808.23 | 3.74 | 20.4 | 9.21 | 755.45 | 4.95 | 1.07 | 148.2 | 25.82 |
| CBSBH02 |  | 42.39° 128.10° |  | 5.82 | 2.42 | 0.21 | 849.5 | 3.86 | 16.55 | 4.64 | 853.10 | 3.46 | 1.04 | 98.49 | 16.41 |
| CBSBH03 |  | 42.39° 128.10° |  | 5.5 | 1.82 | 0.14 | 294.32 | 3.46 | 12.21 | 1.19 | 349.42 | 4.13 | 1.19 | 126.81 | 28.17 |
| DQGZZS | Inner Mongolia, Daqinggou | 42.82° 122.19° | Temperate deciduous needle-leaf forest | 6.58 | 0.5 | 0.05 | 13.56 | 2.95 | 5.22 | 1.34 | 36.09 | 1.71 | 0.83 | 46.49 | 6.99 |
| DQGKY |  | 42.82° 122.20° | Temperate coniferous and broadleaved mixed forest | 6.56 | 0.4 | 0.04 | 11.22 | 3.23 | 5.79 | 0.97 | 175.05 | 1.99 | 1.44 | 41.12 | 6.22 |
| MESCSL | Heilongjiang, Maoershan | 45.15° 127.40° | Temperate secondary weed tress forest | 5.29 | 5.3 | 0.5 | 1742.5 | 13.7 | 7.57 | 10.95 | 889.16 | 3.85 | 1.23 | 263.91 | 32.69 |
| MESMGL |  | 45.24° 127.40° | Temperate Mongolian oak forest | 5.83 | 4.29 | 0.38 | 749.01 | 14.76 | 7.21 | 7.27 | 664.47 | 6.39 | 1.06 | 218.47 | 39.62 |
| MESLYS |  | 45.24° 127.40° | Temperate larch plantation-50 years | 5.67 | 2.11 | 0.21 | 680.54 | 61.23 | 1.67 | 6.27 | 200.43 | 3.76 | 1.18 | 166.64 | 23.73 |
| MESZZS |  | 45.25° 127.40° | Temperate pinus Sylvestris Var. Mongolica plantaiton-40 years | 5.84 | 3.25 | 0.34 | 798.65 | 39.14 | 6.31 | 8.86 | 393.57 | 4.24 | 1.04 | 220.54 | 30.19 |
| LSHJL | Heilongjiang, Liangshui | 47.11° 128.54° | Temperate coniferous and broadleaved mixed forest | 5.51 | 8.67 | 0.66 | 1457.53 | 41.12 | 8.16 | 7.61 | 1135.20 | 5.38 | 1.24 | 306.74 | 49.12 |
| LSHS |  | 47.11° 128.54° | Temperate Korean pine forest | 5.43 | 6.48 | 0.4 | 531.09 | 10.71 | 19.82 | 5.74 | 716.23 | 4.76 | 0.9 | 220.21 | 34.16 |
| DXABH01 | Inner Mongolia, Daxingan | 51.43° 121.26° | Temperate birch forest | 5.23 | 3.54 | 0.18 | 334.16 | 50.19 | 6.85 | 0.58 | 609.83 | 3.99 | 0.58 | 88.27 | 16.15 |
| DXABH02 |  | 51.43° 121.26° |  | 5.1 | 3.44 | 0.16 | 551.45 | 86.36 | 7.92 | 0.45 | 593.23 | 3.56 | 0.55 | 73.55 | 15.67 |
| DXABH03 |  | 51.43° 121.26° |  | 5.63 | 2.59 | 0.15 | 382.4 | 19.33 | 11.49 | 0.47 | 461.37 | 4.49 | 0.76 | 119.68 | 21.41 |
| DXASY01 |  | 51.50° 121.25° | Temperate populus davidiana forest | 5.85 | 3.09 | 0.19 | 973.91 | 15.73 | 7.56 | 1.92 | 458.69 | 6.15 | 1.11 | 186.33 | 43.2 |
| DXASY02 |  | 51.50° 121.25° |  | 5.34 | 2.16 | 0.13 | 906.41 | 28.77 | 7.52 | 0.7 | 334.06 | 4.48 | 0.69 | 105.08 | 27.3 |
| DXASY03 |  | 51.50° 121.25° |  | 5.76 | 3.51 | 0.21 | 984.58 | 15.24 | 12.04 | 0.84 | 459.92 | 4.8 | 1.26 | 191.86 | 47.55 |
| DXALYS01 |  | 51.53° 121.24° | Temperate larch forest | 6.5 | 2.72 | 0.21 | 2468.48 | 35.27 | 3.65 | 1.12 | 449.40 | 3.36 | 0.55 | 184.31 | 42.57 |
| DXALYS02 |  | 51.53° 121.24° |  | 6.05 | 1.84 | 0.14 | 1365.99 | 15.24 | 3.39 | 0.72 | 287.66 | 2.79 | 0.94 | 172.19 | 44.6 |
| DXALYS03 |  | 51.53° 121.24° |  | 5.97 | 1.9 | 0.12 | 4067.83 | 121.01 | 5.88 | 0.73 | 375.00 | 5.33 | 0.57 | 124.91 | 23.85 |
| DXAHJL01 |  | 51.53° 121.24° | Temperate coniferous and broadleaved mixed forest | 6.31 | 3.14 | 0.22 | 843.6 | 137.77 | 10.67 | 0.79 | 718.40 | 18.3 | 0.79 | 195.83 | 41.59 |
| DXAHJL02 |  | 51.53° 121.24° |  | 6.1 | 1.73 | 0.11 | 443.84 | 25.52 | 4.23 | 1.3 | 274.41 | 3.93 | 0.63 | 149.68 | 27.59 |
| DXAHJL03 |  | 51.53° 121.24° |  | 6.38 | 1.68 | 0.09 | 622.78 | 19.9 | 3.73 | 0.79 | 392.34 | 3.77 | 0.76 | 204.87 | 19.61 |

a MBC: soil microbial biomass carbon.

b EK, ENa, ECa and EMg represents soil exchangeable K^+^, Na^+^, Ca^2+^ and Mg^2+^ contents, respectively.
