# Supplementary material for: Biogeographic Distribution Patterns of Bacteria in Typical Chinese Forest Soils
Source: Front Microbiol. 2016 Jul 13;7:1106. doi: 10.3389/fmicb.2016.01106 (PMC4942481; doi:10.3389/fmicb.2016.01106)
Supplement: Supplementary file 2 [file Table_2.DOC]

Table S2 All bacterial taxa present in all sampled soils and their relative abundance.

| Taxa | Relative abundance | Taxa | Relative abundance |
| --- | --- | --- | --- |
| *Actinobacteria* | 21.91393% | *GN02* | 0.00343% |
| *Acidobacteria* | 18.47293% | *NKB19* | 0.00306% |
| *Alphaproteobacteria* | 17.29648% | *GN04* | 0.00266% |
| *Verrucomicrobia* | 8.68228% | *NC10* | 0.00261% |
| *Planctomycetes* | 6.74748% | *Synergistetes* | 0.00259% |
| *Chloroflexi* | 4.73685% | *FBP* | 0.00201% |
| *Betaproteobacteria* | 3.88829% | *OP11* | 0.00161% |
| *Deltaproteobacteria* | 3.61119% | *SBR1093* | 0.00156% |
| *Gammaproteobacteria* | 3.28364% | *Fusobacteria* | 0.00135% |
| *Gemmatimonadetes* | 2.13467% | *MVP-21* | 0.00116% |
| *Nitrospirae* | 1.79988% | *[Thermi]* | 0.00107% |
| *Bacteroidetes* | 1.37480% | *GOUTA4* | 0.00066% |
| *AD3* | 1.12468% | *WS4* | 0.00054% |
| *Firmicutes* | 0.78318% | *Lentisphaerae* | 0.00045% |
| *WPS-2* | 0.73416% | *OP8* | 0.00032% |
| *WS3* | 0.38825% | *WWE1* | 0.00026% |
| *Other Proteobacteria* | 0.33914% | *SAR406* | 0.00017% |
| *Armatimonadetes* | 0.26645% | *PAUC34f* | 0.00017% |
| *Archaea* | 0.25981% | *MVS-104* | 0.00017% |
| *TM6* | 0.11948% | *Deferribacteres* | 0.00017% |
| *Cyanobacteria* | 0.09992% | *OC31* | 0.00013% |
| *Elusimicrobia* | 0.09872% | *SC4* | 0.00011% |
| *Chlamydiae* | 0.08488% | *Thermotogae* | 0.00011% |
| *TM7* | 0.08199% | *ZB3* | 0.00006% |
| *Chlorobi* | 0.04647% | *Kazan-3B-28* | 0.00006% |
| *GAL15* | 0.04595% | *AC1* | 0.00006% |
| *OD1* | 0.02914% | *WS1* | 0.00006% |
| *OP3* | 0.02408% | *SR1* | 0.00006% |
| *BRC1* | 0.01566% | *[Caldithrix]* | 0.00004% |
| *FCPU426* | 0.00799% | *Caldiserica* | 0.00004% |
| *WS2* | 0.00765% | *WS5* | 0.00004% |
| *Fibrobacteres* | 0.00698% | *OP9* | 0.00004% |
| *Spirochaetes* | 0.00658% | *OP1* | 0.00002% |
| *BHI80-139* | 0.00643% | *Caldithrix* | 0.00002% |
| *Tenericutes* | 0.00356% | *Unclassified* | 1.44953% |
