# Supplementary material for: Biogeographic Distribution Patterns of Bacteria in Typical Chinese Forest Soils
Source: Front Microbiol. 2016 Jul 13;7:1106. doi: 10.3389/fmicb.2016.01106 (PMC4942481; doi:10.3389/fmicb.2016.01106)
Supplement: Supplementary file 3 [file Table_3.DOC]

Table S3. The linear regression between relative abundance of *Acidobacteria* subgroups and soil/site properties. *r* and *P* represent the coefficient of the linear regression and the significance value, respectively. Values in bold indicate significant correlations (*P* < 0.01). MAT and MAP represent mean annual temperature and mean annual precipitation; EK, ENa, ECa and EMg represent soil exchangeable K^+^, Na^+^, Ca^2+^ and Mg^2+^ contents, respectively; TC, TN, TP and AP represent soil total carbon, total nitrogen, total phosphorus and available phosphorus contents, respectively; NH_4_^+^ and NO_3_^-^ represent soil extractable NH_4_^+^ and NO_3_^-^ levels, respectively; MBC represents soil microbial biomass carbon.

|  |  | Latitude | MAT | MAP | pH | ENa | EK | ECa | EMg | TC | TN | TP | AP | NH_4_^+^ | NO_3_^−^ | MBC |
| --- | --- | --- | --- | --- | --- | --- | --- | --- | --- | --- | --- | --- | --- | --- | --- | --- |
| *GP1* | *r* | **-0.455** | **0.466** | **0.510** | **-0.803** | 0.017 | **-0.639** | **-0.797** | **-0.723** | **-0.378** | **-0.394** | **-0.410** | -0.203 | -0.227 | **-0.405** | 0.123 |
|  | *P* | 0.000 | 0.000 | 0.000 | 0.000 | 0.857 | 0.000 | 0.000 | 0.000 | 0.000 | 0.000 | 0.000 | 0.029 | 0.015 | 0.000 | 0.191 |
| *GP2* | *r* | **-0.399** | **0.407** | **0.486** | **-0.809** | -0.027 | **-0.633** | **-0.756** | **-0.693** | **-0.299** | **-0.341** | **-0.415** | -0.230 | -0.153 | **-0.387** | 0.076 |
|  | *P* | 0.000 | 0.000 | 0.000 | 0.000 | 0.774 | 0.000 | 0.000 | 0.000 | 0.001 | 0.000 | 0.000 | 0.013 | 0.102 | 0.000 | 0.420 |
| *GP3* | *r* | -0.117 | 0.166 | **0.267** | **-0.527** | -0.036 | **-0.507** | **-0.511** | **-0.467** | **-0.298** | -0.186 | **-0.286** | -0.166 | -0.209 | **-0.300** | 0.166 |
|  | *P* | 0.213 | 0.076 | 0.004 | 0.000 | 0.704 | 0.000 | 0.000 | 0.000 | 0.001 | 0.047 | 0.002 | 0.076 | 0.025 | 0.001 | 0.077 |
| *GP4* | *r* | **0.568** | **-0.547** | **-0.653** | **0.681** | 0.089 | **0.613** | **0.625** | **0.607** | 0.154 | 0.152 | **0.251** | 0.228 | -0.077 | 0.131 | -0.155 |
|  | *P* | 0.000 | 0.000 | 0.000 | 0.000 | 0.346 | 0.000 | 0.000 | 0.000 | 0.100 | 0.106 | 0.007 | 0.014 | 0.414 | 0.164 | 0.097 |
| *GP5* | *r* | 0.008 | -0.046 | -0.077 | **0.253** | 0.180 | 0.203 | 0.178 | 0.147 | -0.007 | 0.050 | -0.008 | -0.090 | 0.052 | -0.062 | -0.051 |
|  | *P* | 0.933 | 0.623 | 0.416 | 0.006 | 0.054 | 0.029 | 0.057 | 0.117 | 0.943 | 0.596 | 0.931 | 0.339 | 0.580 | 0.509 | 0.587 |
| *GP6* | *r* | **0.314** | **-0.355** | **-0.391** | **0.771** | -0.076 | **0.645** | **0.745** | **0.710** | **0.400** | **0.407** | **0.418** | **0.251** | **0.335** | **0.473** | -0.096 |
|  | *P* | 0.001 | 0.000 | 0.000 | 0.000 | 0.419 | 0.000 | 0.000 | 0.000 | 0.000 | 0.000 | 0.000 | 0.007 | 0.000 | 0.000 | 0.309 |
| *GP7* | *r* | **0.574** | **-0.541** | **-0.533** | **0.390** | 0.051 | 0.187 | **0.355** | **0.339** | -0.039 | 0.075 | 0.132 | 0.033 | -0.206 | -0.053 | 0.003 |
|  | *P* | 0.000 | 0.000 | 0.000 | 0.000 | 0.585 | 0.045 | 0.000 | 0.000 | 0.678 | 0.425 | 0.159 | 0.727 | 0.027 | 0.577 | 0.978 |
| *GP9* | *r* | -0.134 | 0.086 | 0.085 | **0.455** | -0.232 | 0.228 | **0.577** | **0.517** | **0.610** | **0.471** | **0.621** | **0.312** | **0.533** | **0.792** | -0.099 |
|  | *P* | 0.154 | 0.362 | 0.366 | 0.000 | 0.013 | 0.014 | 0.000 | 0.000 | 0.000 | 0.000 | 0.000 | 0.001 | 0.000 | 0.000 | 0.291 |
| *GP10* | *r* | -0.055 | 0.034 | 0.008 | **0.420** | -0.117 | 0.139 | **0.439** | **0.279** | **0.358** | **0.312** | **0.529** | 0.131 | **0.308** | **0.478** | -0.049 |
|  | *P* | 0.562 | 0.721 | 0.929 | 0.000 | 0.213 | 0.138 | 0.000 | 0.003 | 0.000 | 0.001 | 0.000 | 0.164 | 0.001 | 0.000 | 0.604 |
| *GP11* | *r* | -0.033 | 0.009 | -0.107 | **0.515** | 0.189 | **0.408** | **0.506** | **0.387** | **0.292** | **0.276** | 0.173 | -0.095 | **0.272** | **0.323** | -0.052 |
|  | *P* | 0.723 | 0.921 | 0.254 | 0.000 | 0.043 | 0.000 | 0.000 | 0.000 | 0.002 | 0.003 | 0.065 | 0.312 | 0.003 | 0.000 | 0.581 |
| *GP13* | *r* | **-0.418** | **0.458** | **0.599** | **-0.665** | -0.012 | **-0.521** | **-0.617** | **-0.594** | **-0.361** | **-0.333** | **-0.304** | -0.178 | **-0.325** | **-0.295** | 0.009 |
|  | *P* | 0.000 | 0.000 | 0.000 | 0.000 | 0.898 | 0.000 | 0.000 | 0.000 | 0.000 | 0.000 | 0.001 | 0.057 | 0.000 | 0.001 | 0.922 |
| *GP17* | *r* | 0.078 | -0.079 | **-0.255** | **0.738** | 0.114 | **0.605** | **0.773** | **0.615** | **0.518** | **0.467** | **0.433** | 0.060 | **0.334** | **0.605** | -0.047 |
|  | *P* | 0.407 | 0.400 | 0.006 | 0.000 | 0.223 | 0.000 | 0.000 | 0.000 | 0.000 | 0.000 | 0.000 | 0.521 | 0.000 | 0.000 | 0.618 |
| *GP18* | *r* | 0.022 | -0.024 | -0.181 | **0.636** | 0.036 | **0.501** | **0.742** | **0.601** | **0.586** | **0.443** | **0.537** | 0.189 | **0.345** | **0.733** | -0.076 |
|  | *P* | 0.819 | 0.800 | 0.053 | 0.000 | 0.702 | 0.000 | 0.000 | 0.000 | 0.000 | 0.000 | 0.000 | 0.043 | 0.000 | 0.000 | 0.418 |
| *GP21* | *r* | -0.069 | 0.054 | 0.017 | 0.020 | -0.029 | -0.039 | 0.027 | -0.084 | -0.043 | -0.077 | -0.091 | -0.087 | 0.043 | -0.092 | 0.026 |
|  | *P* | 0.465 | 0.564 | 0.859 | 0.835 | 0.761 | 0.677 | 0.779 | 0.372 | 0.650 | 0.415 | 0.333 | 0.352 | 0.649 | 0.328 | 0.782 |
| *GP22* | *r* | 0.021 | -0.058 | -0.130 | **0.485** | 0.076 | **0.410** | **0.521** | **0.328** | **0.292** | 0.236 | 0.234 | -0.050 | **0.254** | **0.241** | 0.028 |
|  | *P* | 0.826 | 0.537 | 0.166 | 0.000 | 0.421 | 0.000 | 0.000 | 0.000 | 0.002 | 0.011 | 0.012 | 0.593 | 0.006 | 0.010 | 0.770 |
| *GP23* | *r* | 0.021 | -0.006 | -0.054 | -0.037 | -0.075 | -0.079 | -0.055 | -0.031 | -0.071 | -0.073 | -0.060 | -0.042 | -0.041 | -0.057 | -0.056 |
|  | *P* | 0.827 | 0.953 | 0.565 | 0.694 | 0.427 | 0.400 | 0.559 | 0.739 | 0.449 | 0.437 | 0.522 | 0.656 | 0.662 | 0.547 | 0.550 |
| *GP25* | *r* | 0.108 | -0.083 | -0.234 | **0.577** | 0.170 | **0.311** | **0.516** | **0.443** | **0.263** | **0.265** | **0.241** | 0.000 | 0.080 | **0.353** | -0.162 |
|  | *P* | 0.250 | 0.375 | 0.012 | 0.000 | 0.070 | 0.001 | 0.000 | 0.000 | 0.005 | 0.004 | 0.009 | 0.999 | 0.392 | 0.000 | 0.083 |
