# Supplementary material for: Biogeographic Distribution Patterns of Bacteria in Typical Chinese Forest Soils
Source: Front Microbiol. 2016 Jul 13;7:1106. doi: 10.3389/fmicb.2016.01106 (PMC4942481; doi:10.3389/fmicb.2016.01106)
Supplement: Supplementary file 4 [file Image_1.PDF]

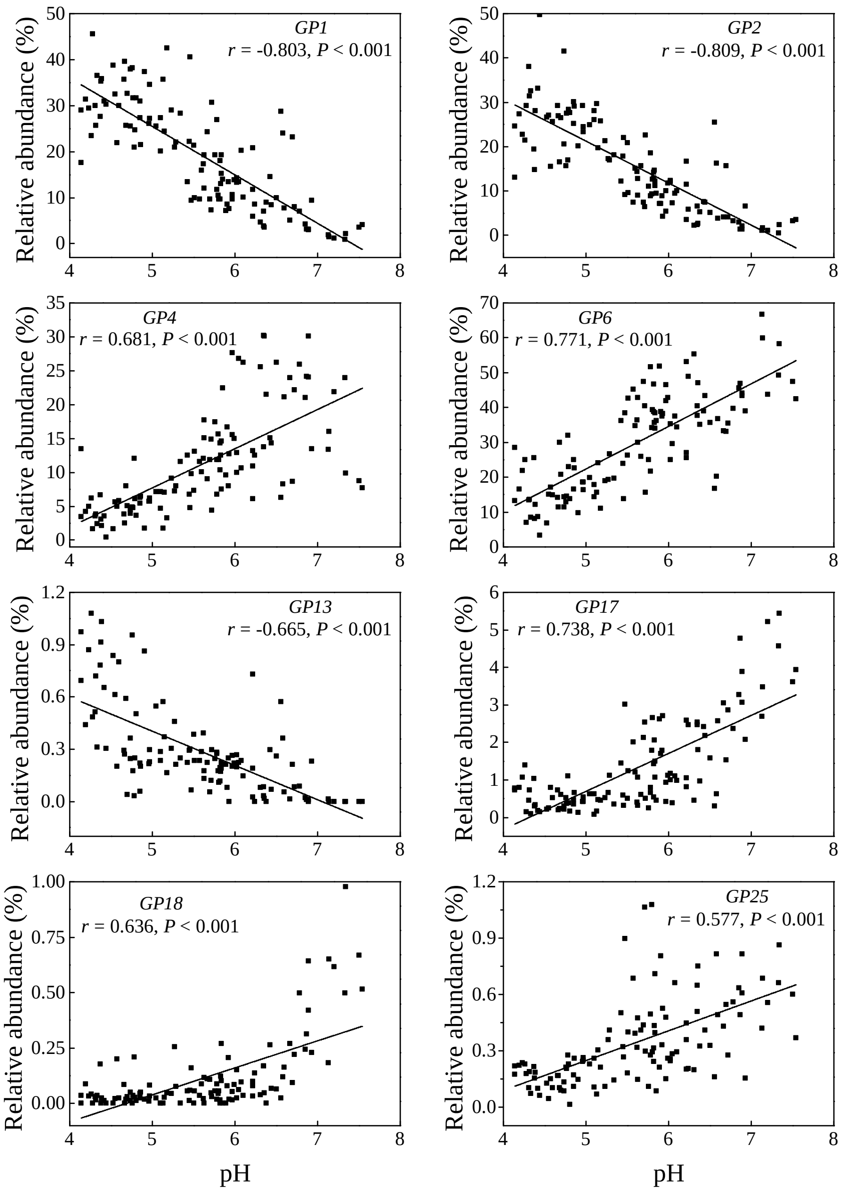

Figure S1. Relationship between the relative abundance of dominant subgroups of *Acidobacteria* and soil pH.
