# Supplementary material for: Biogeographic Distribution Patterns of Bacteria in Typical Chinese Forest Soils
Source: Front Microbiol. 2016 Jul 13;7:1106. doi: 10.3389/fmicb.2016.01106 (PMC4942481; doi:10.3389/fmicb.2016.01106)
Supplement: Supplementary file 7 [file Image_4.PDF]

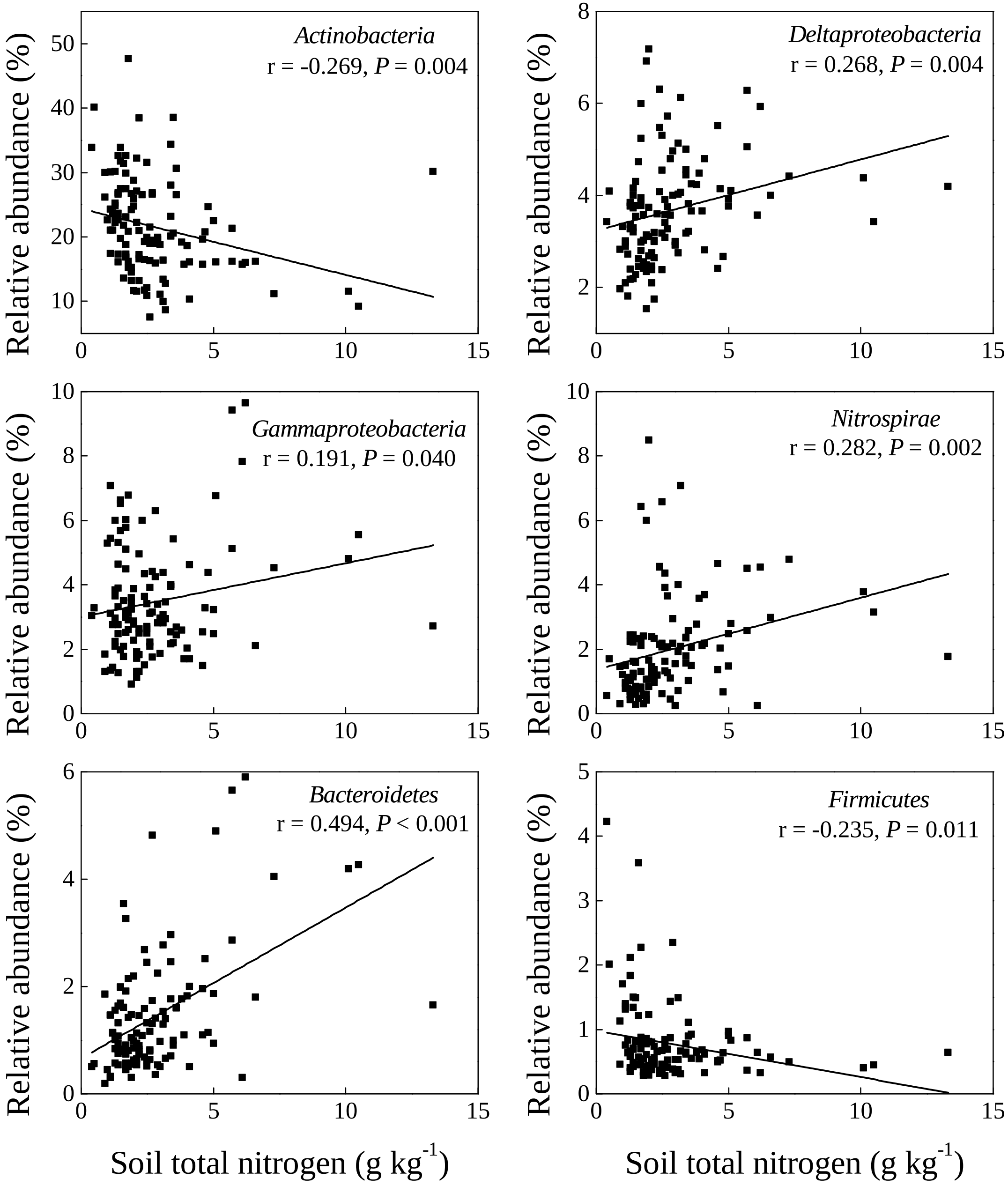

Figure S4. Relationship between relative abundance of dominant bacterial groups and soil total nitrogen.
