# Supplementary figures and images for: Biogeographic Distribution Patterns of Bacteria in Typical Chinese Forest Soils
Source: Front Microbiol. 2016 Jul 13;7:1106. doi: 10.3389/fmicb.2016.01106 (PMC4942481; doi:10.3389/fmicb.2016.01106)

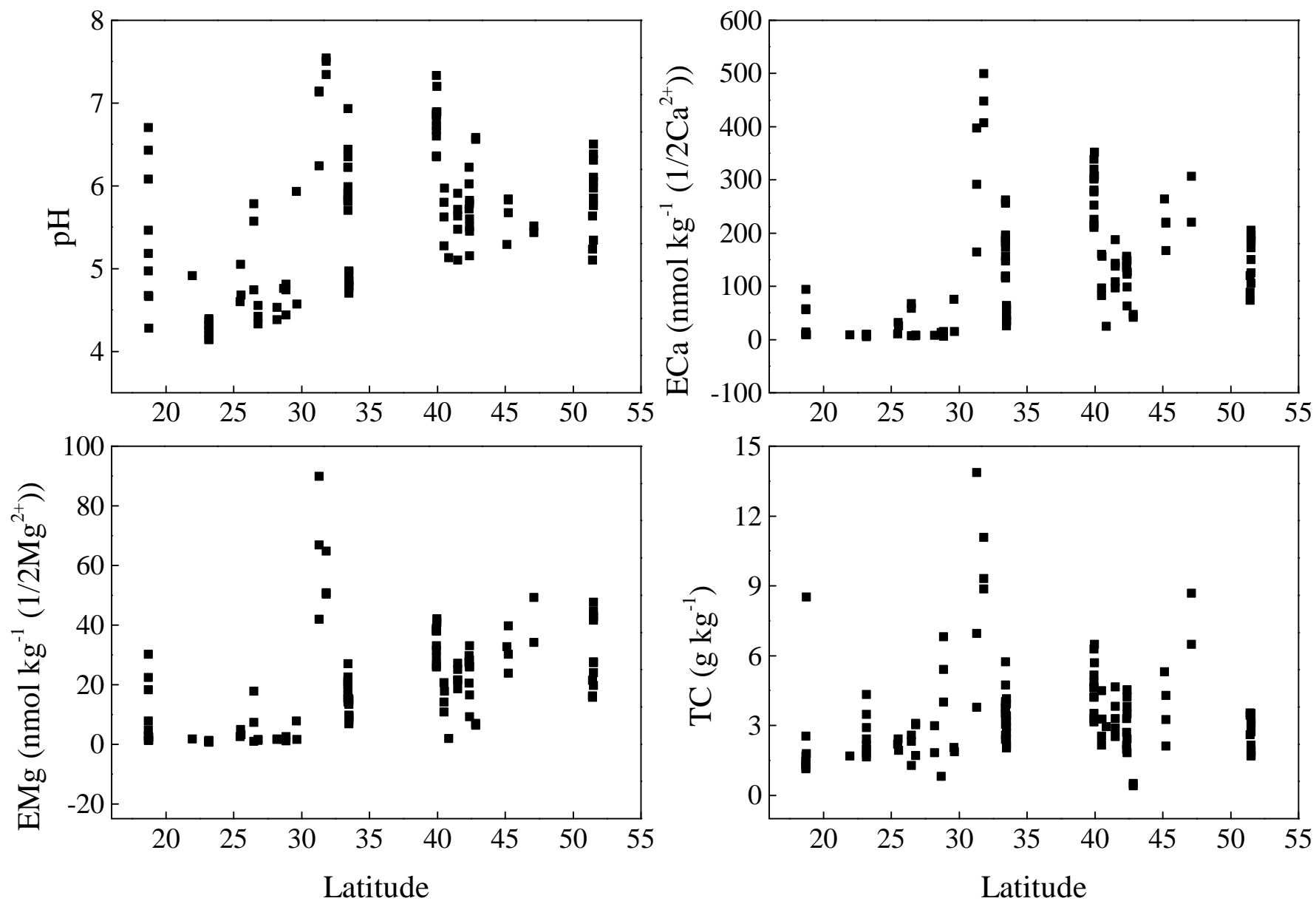

Figure S5. The changes of soil parameters along the latitude of sampling sites.

Supplement: Supplementary file 8 [file Image_5.PDF]
