# Supplementary material for: Biogeographic Distribution Patterns of Bacteria in Typical Chinese Forest Soils
Source: Front Microbiol. 2016 Jul 13;7:1106. doi: 10.3389/fmicb.2016.01106 (PMC4942481; doi:10.3389/fmicb.2016.01106)
Supplement: Supplementary file 9 [file Image_6.PDF]

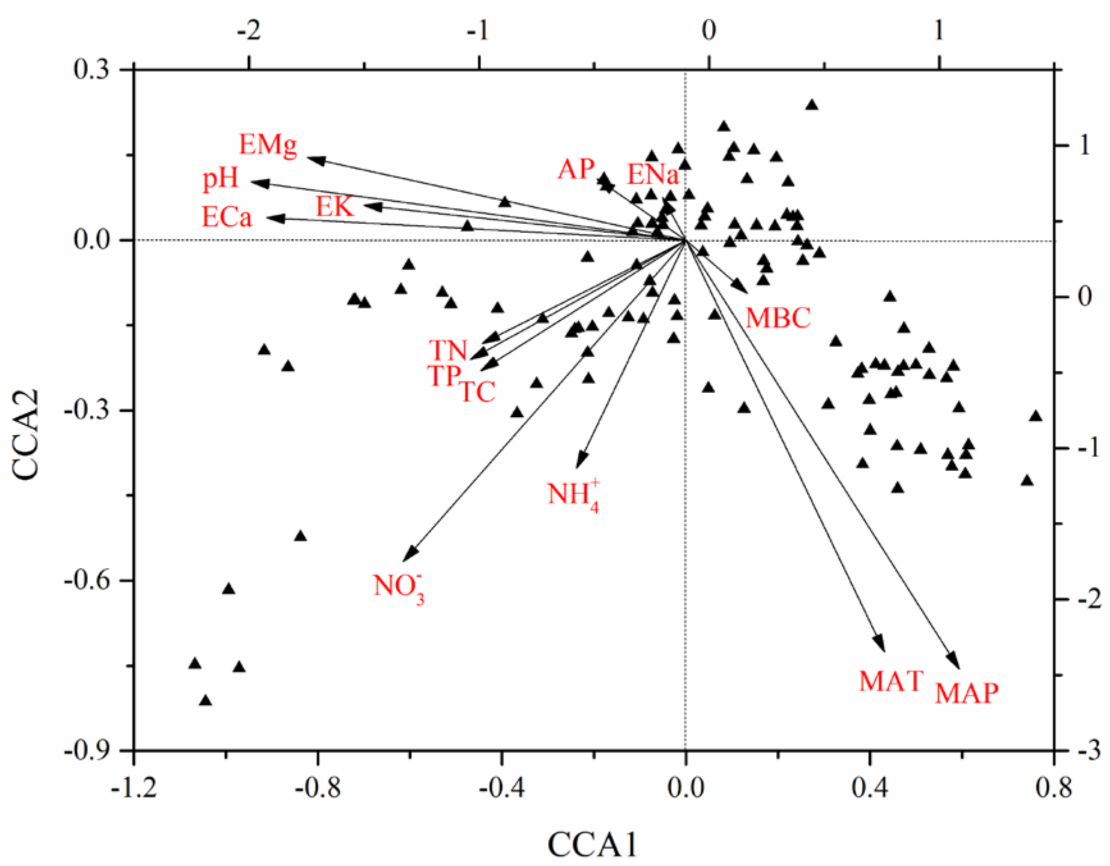

Figure S6. Canonical correspondence analysis (CCA) of the bacterial community composition and environmental variables. All the 115 samples were displayed with black triangles in the plot scaled by top and right axises.
